# LONP1 and ClpP cooperatively regulate mitochondrial proteostasis for cancer cell survival

**DOI:** 10.1038/s41389-021-00306-1

**Published:** 2021-02-26

**Authors:** Yu Geon Lee, Hui Won Kim, Yeji Nam, Kyeong Jin Shin, Yu Jin Lee, Do Hong Park, Hyun-Woo Rhee, Jeong Kon Seo, Young Chan Chae

**Affiliations:** 1 School of Life Sciences, Ulsan National University of Science and Technology (UNIST), Ulsan, 44919 Republic of Korea; 2 Department of Chemistry, Seoul National University, Seoul, 08826 Republic of Korea

**Keywords:** Oncogenes, Protein folding

## Abstract

Mitochondrial proteases are key components in mitochondrial stress responses that maintain proteostasis and mitochondrial integrity in harsh environmental conditions, which leads to the acquisition of aggressive phenotypes, including chemoresistance and metastasis. However, the molecular mechanisms and exact role of mitochondrial proteases in cancer remain largely unexplored. Here, we identified functional crosstalk between LONP1 and ClpP, which are two mitochondrial matrix proteases that cooperate to attenuate proteotoxic stress and protect mitochondrial functions for cancer cell survival. LONP1 and ClpP genes closely localized on chromosome 19 and were co-expressed at high levels in most human cancers. Depletion of both genes synergistically attenuated cancer cell growth and induced cell death due to impaired mitochondrial functions and increased oxidative stress. Using mitochondrial matrix proteomic analysis with an engineered peroxidase (APEX)-mediated proximity biotinylation method, we identified the specific target substrates of these proteases, which were crucial components of mitochondrial functions, including oxidative phosphorylation, the TCA cycle, and amino acid and lipid metabolism. Furthermore, we found that LONP1 and ClpP shared many substrates, including serine hydroxymethyltransferase 2 (SHMT2). Inhibition of both LONP1 and ClpP additively increased the amount of unfolded SHMT2 protein and enhanced sensitivity to SHMT2 inhibitor, resulting in significantly reduced cell growth and increased cell death under metabolic stress. Additionally, prostate cancer patients with higher LONP1 and ClpP expression exhibited poorer survival. These results suggest that interventions targeting the mitochondrial proteostasis network via LONP1 and ClpP could be potential therapeutic strategies for cancer.

## Introduction

Mitochondria are intracellular organelles that produce the majority of energy in cells by synthesizing ATP via oxidative phosphorylation. Beyond cellular bioenergetics, mitochondria have many other functions including regulation of redox balance, metabolite biosynthesis, regulation of calcium homeostasis, and modulation of cell death pathways^1,2^. Therefore, mitochondria homeostasis is tightly regulated. Cancer cells rely heavily on mitochondria to meet their high energy demands and generate macromolecules used as building blocks to fuel rapid cancer cell proliferation^1,3,4^. Additionally, mitochondria serve as a central signaling hub that integrates extracellular and intracellular signaling pathways and nutrient status controlling epigenetics, stemness, cell cycle regulation, and apoptosis and are thus involved in all phases of cancer initiation, progression, and metastasis^5–7,8^. Therefore, mitochondria have emerged as a therapeutic target for novel anticancer agents^9,10^.

As cancer cells are continuously exposed to various cytotoxic stressors in harsh tumor microenvironments, including hypoxia, nutrient deprivation, and oxidative stress, mitochondria must adapt to changes and buffer stress conditions to promote cancer cell proliferation and survival^11,12^. Indeed, mitochondrial stress responses are linked to alterations in mitochondrial functions required for tumor progression via adaptations to changing metabolic demands, regulation of cell death pathways, and contributions to chemoresistance^4^. One well-characterized cytoprotective mitochondrial stress response pathway is the protein quality control system, which maintains mitochondrial protein homeostasis by degrading misfolded and aggregated proteins to buffer proteotoxic stress associated with stressful conditions^13^. Different from the ubiquitin/proteasome system for cytosolic protein homeostasis, mitochondrial chaperone and protease proteins are crucial for mitochondrial proteostasis, are overexpressed in most tumor types, and are involved in metabolic reprogramming that allows evasion of apoptosis and increased survival^14,15,16^. However, the molecular mechanisms regulating mitochondrial proteostasis and its exact function in cancer remain largely unknown.

Among the effectors of mitochondrial proteostasis, mitochondrial matrix serine proteases Lon protease (LONP1) and caseinolytic peptidase P (ClpP) are key inducers of mitochondrial protein quality control for the clearance of misfolded or damaged proteins, which is necessary for maintaining mitochondrial functions. Elevated activities of these two proteases are correlated with tumor development and progression^17,18,15^. These proteases may modulate cancer cell viability, reactive oxygen species (ROS) levels, and metabolic reprogramming under hypoxia, oxidative stress, or starvation^14^. Consequently, inhibition or hyperactivation of these proteases may be a potential therapeutic strategy for cancer^18^. Although LONP1 and ClpP exert similar actions in cancer, their regulatory mechanisms and potential interconnections remain largely unexplored. Here, we identified the specific target substrates of each protease and discovered functional crosstalk through which LONP1 and ClpP cooperatively modulate aspects of mitochondrial proteostasis that are crucial for cancer cell growth and survival. These findings suggest that targeting the interplay between LONP1 and ClpP could be a potential therapeutic strategy for cancer.

## Materials and methods

### Antibodies and reagents

Antibodies against ClpP (Abcam, Cambridge, MA, USA, #ab124822), LONP1 (Novus Biologicals, Centennial, CO, USA, #NBP1-81734), beta-actin (GeneTex, San Antonio, TX, USA, #GTX109639), cleaved PARP (Cell Signaling, Danvers, MA, USA, #9541), PARP (Cell Signaling, #9532), Thr172-phosphorylated AMPK (Cell Signaling, #2531), AMPK (Cell Signaling, #2630), LC3B (Cell Signaling, #2775), HRP-conjugated streptavidin (Sigma Aldrich, St. Louis, MO, USA #RABHRP3), SHMT2 (Atlas Antibodies, Bromma, Stockholm, Sweden, #HPA020549), SHMT1 (Santa Cruz Biotechnology, Santa Cruz, CA, USA, sc-365203), Ser555-phosphorylated ULK1 (Cell Signaling, #5869 S), ULK1 (Cell Signaling, #9661), p62/SQSTM1 antibodies (Santa Cruz Biotechnology, sc-28359), TUFM (Abcam, #ab175199), PDK1 (Abcam, #ab110025), ATP5B (Abcam, #ab14730), FH (Abcam, #ab191367), SSBP1 (Proteintech, Chicago, IL, USA, #12212-1-AP), VDAC (Abcam, #ab14734), and alpha-tubulin (GeneTex, #GTX112141) were used for western blotting. (+)-SHIN1 was from MedChmexpress (Princeton, NJ, USA, #HY-112066A). MitoTracker Red (#M7512) and MitoSOX Red (#M36008) were from Invitrogen. Sequencing-grade trypsin, sodium azide, sodium ascorbate, and Trolox were from Sigma Aldrich (St. Louis, MO, USA). Plasmids for APEX fusion constructs for targeting mitochondria (mito-APEX) and biotin-phenol were provided by Prof. Hyun-Woo Rhee (Seoul National University, Seoul, Korea).

### Cell culture

Human prostate adenocarcinoma LNCaP, PC3, DU145, 22RV1, C4-2, and C4-2B cells; normal prostate epithelial RWPE1 cells; benign prostatic hyperplasia epithelial BPH-1 cells; human glioblastoma LN229 cells; and human colorectal carcinoma HCT116 and SW480 cells; human embryonic kidney 293 (HEK-293) cells were obtained from the American Type Culture Collection (ATCC, Manassas, VA, USA). All cell lines were maintained in culture according to the supplier’s recommendations. For glucose starvation experiments, cells were cultured in glucose deprived-RPMI 1640 medium (Gibco, Gaithersburg, MD, USA, #11879-020) supplemented with glucose (0.5 or 1 mM) and 2% fetal bovine serum. Cells were incubated for 24 h.

### Transfections

To knock down LONP1 and ClpP by siRNA, cancer cell lines were transfected with non-targeting siRNA (Dharmacon, Lafayette, CO, USA, #D-001210), siRNA pools targeting LONP1 (Dharmacon, #L-003979-00-0020), or custom-prepared ClpP-directed siRNA with the sequence GUUUGGCAUCUUAGACAAGGUUCUGUU. siRNAs were transfected at 20 nM in the presence of lipofectamine RNAiMAX (Invitrogen, Carlsbad, CA, USA) at a 1:1.5 ratio. Cells were incubated for 48 h, validated for target protein knockdown by western blotting, and processed for subsequent experiments.

### Quantitative RT-PCR analysis

Total RNA was extracted with TRIzol (Ambion, Austin, TX, USA) according to the manufacturer’s instructions. RNA (2 μg) was reverse-transcribed using a high-capacity cDNA reverse transcription kit (Applied Biosystems, Foster City, CA, USA). Equal amounts of cDNA for each sample were mixed with Prime Q-Mastermix (Genet Bio, Chungnam, Korea). qRT-PCR reactions were performed in a Light Cycler 480 system (Roche Applied Science, Mannheim, Germany). The following real-time quantitative PCR primers were used: LONP1: forward, 5′-ATGGAGGACGTCAAGAAACG-3′ and reverse, 5′-GACGCTGAAGCGGAAGTACTC-3′; ClpP: forward, 5′-TTGCCAGCCTTGTTATCGCA-3′ and reverse, 5′-GGTTGAGGATGTACTGCATCG-3′; SHMT2: forward, 5′-TCGGAGGGTTATCCTGGCAA-3′ and reverse, 5′-TTTAGGGCCACAGCTACTGC-3′; GAPDH: forward, 5′-GAAGGTGAAGGTCGGAGTC-3′ and reverse, 5′-GAAGATGGTGATGGGATTTC-3′. ATF4: forward, 5′-CCAACAACAGCAAGGAGGAT-3′ and reverse, 5′-GGGGCAAAGAGATCACAAGT-3′; ATF6: forward, 5′-TGACAAAGCCCTGATGGTGCTA-3′ and reverse, 5′-TGTTCCAGAGCACCCTGAAGAA-3′; CHOP: forward, 5′-GGAGAACCAGGAAACGGAAAC-3′ and reverse, 5′-TCTCCTTCATGCGCTGCTTT-3′. mRNA levels were normalized to GAPDH mRNA level as a control and calculated according to the ΔΔCt method.

### Protein analysis

Cells were washed twice with cold phosphate-buffered saline (PBS) and lysed with RIPA buffer (150 mM NaCl, 1.0% Triton X-100, 0.5% sodium deoxycholate, 0.1% SDS, 50 mM Tris, pH 8.0) containing protease and phosphatase inhibitor cocktail (Invitrogen). Equal amounts of protein lysates were separated by SDS gel electrophoresis, transferred to a PVDF membrane, and incubated with primary antibodies. After incubation with horseradish peroxidase-conjugated secondary antibodies (Santa Cruz Biotechnology), protein bands were visualized by chemiluminescence. Densitometry was performed using ImageJ software.

### Mitochondrial DNA levels

DNA was isolated from LNCaP or DU145 cells using a DNA Purification Kit (Macherey-Nagel, Duren, Germany). The ratio of mitochondrial DNA (mtDNA) to genomic DNA was determined by performing qPCR with nDNA β2-microglobulin (forward, TGCTGTCTCCATGTTTGATGTATCT; reverse, TCTCTGCTCCCCACCTCTAAGT), MT-ATP6 (forward, TAGCCATACACAACACTAAAGGACGA; reverse, GGGCATTTTTAATCTTAGAGCGAAA), and MT-CYB (forward, ATCACTCGAGACGTAAATTATGGCT; reverse, TGAACTAGGTCTGTCCCAATGTATG) primers. All primer pairs were run in individual reactions. The final mtDNA/nucDNA ratio for each sample was calculated by averaging the ratio obtained from each primer pair. Expression data were analyzed with the ΔΔCt method.

### Mitochondrial protein folding

Mitochondrial protein folding assay was carried out as previously described^19^. LNCaP or DU145 cells were transfected with control non-targeting siRNA or LONP1 and/or ClpP-directed siRNA for 48 h. Mitochondrial fractions were prepared using a mitochondria isolation kit (BioVision Milpitas, CA, USA #K256-25) following the manufacturer’s recommendations. Briefly, cells were mechanically disrupted by 70 strokes in a Dounce homogenizer in isolation buffer A containing protease inhibitor cocktail. Cell debris and nuclei were removed by centrifugation at 700 *g* for 10 min, and mitochondrial fractions were precipitated by centrifugation at 3000 *g* for 25 min. To obtain highly enriched mitochondrial fractions, samples were subject to another round of centrifugation at 12,000 *g* for 10 min in isolation buffer C, and the final pellet was used as an isolated mitochondrial fraction. Mitochondrial fractions were suspended in an equal volume of mitochondrial fractionation buffer containing increasing concentrations of CHAPS (0, 1, or 2.5%). Samples were incubated for 20 min on ice, and detergent-insoluble protein aggregates were recovered by centrifugation (20,000 *g*) for 20 min. Pelleted proteins were separated by SDS gel electrophoresis and visualized by SYPRO Ruby staining (Invitrogen).

### Colony formation

LNCaP or DU145 cells (5 × 10^3^ cells per well) transfected with control non-targeting siRNA or LONP1 and/or ClpP-directed siRNA for 48 h or treated with vehicle or SHIN1 (0–10 μM) were plated in triplicate in 6-multiwell plates. After 7–10 days, colonies were washed in PBS and fixed/stained for 30 min in 0.5% w/v crystal violet/methanol. Plates were rinsed in tap water and dried before scoring. Macroscopically visible colonies were manually counted.

### ATP production

ATP concentration in cells was measured using an ATP Assay kit (BioChain, Newark, CA, USA #Z5030041). Briefly, cells (1 × 10^4^) were seeded in 96-well white plates in complete media and incubated for 1 day at 37 °C. Culture media was removed, and a reaction mixture was added immediately. To quantify ATP production, luminescence was measured within 1 min. Individual values were normalized by cell number per well.

### ROS production

Mitochondrial superoxide production was visualized by fluorescence microscopy. Briefly, cells (2 × 10^4^) transfected with control non-targeting siRNA or LONP1 and/or ClpP-directed siRNA for 48 h were reseeded on high optical quality 8-well μ-slides (Ibidi) and stained with MitoSOX Red mitochondrial superoxide indicator (5 μM, 10 min) in complete medium. Stained cells were imaged with a ×40 objective on a Nikon TE300 inverted time-lapse microscope equipped with a video system containing an Evolution QEi camera and time-lapse video cassette recorder. The atmosphere was equilibrated to 37 °C and 5% CO_2_ in an incubation chamber. Phase and red fluorescence (TRITC filter cube, excitation wavelength: 532–554 nm, emission wavelength: 570–613 nm) images were captured. To quantify superoxide levels, an equal number of stained cells (1 × 10^4^ in 100 μL) were suspended in PBS, and fluorescence (Ex/Em: 510/580 nm) was measured immediately.

### Proteome mapping for mitochondrial proteins by APEX-mediated biotinylation

Labeling of the mitochondrial matrix proteome in cells was performed as previously described^20,21^. Briefly, mito-APEX was transfected into LNCaP cells. After 24 h, cells were transfected with control non-targeting siRNA or LONP1 or ClpP-directed siRNA for 48 h. The medium was changed to 1 mL serum-free medium containing biotin-phenol (500 μM) for 30 min at 37 °C. Then, H_2_O_2_ (final concentration, 1 mM) was added for 1 min to initiate biotinylation. The reaction was terminated by the addition of a quencher solution containing 10 mM sodium azide, 10 mM sodium ascorbate, and 5 mM Trolox in Dulbecco’s PBS (DPBS). For streptavidin blotting, cells were lysed with RIPA buffer containing quencher solution and protease and phosphatase inhibitor cocktail (Invitrogen). Lysates were transferred to an e-tube and clarified by centrifugation at 15,000 *g* for 10 min at 4 °C before separation on a 10% SDS-PAGE gel. The gel was transferred to a nitrocellulose membrane and blocked with 2% (w/v) dialyzed BSA (dBSA) in TBST at room temperature for 1 h. The blots were immersed in streptavidin-HRP in 2% dialyzed BSA in TBST at room temperature for 30–60 min and then rinsed with TBST before development and imaging by chemiluminescence. For imaging analysis, cells were stained with 100 nM MitoTracker Red Reagent (Invitrogen) for 30 min at 37 °C according to the manufacturer’s instructions. Cells were then fixed with 4% paraformaldehyde solution in DPBS for 15 min, washed with DPBS three times, and permeabilized with cold MeOH for 5 min. After three washes with DPBS, cells were blocked for 1 h with dBSA in DPBS. Cells were stained with anti-V5 (Invitrogen) in blocking buffer for 1 h to detect mito-APEX expression. After washing four times with TBST, cells were incubated with secondary goat anti-mouse-AF antibody (Invitrogen) in blocking buffer for 1 h. Samples were washed with PBS four times and maintained in DPBS at room temperature for imaging. Imaging was performed in confocal mode using a confocal microscope (ZEISS, LSM780).

For proteomic mapping by liquid chromatography-mass spectrometry analysis, cell lysates were sonicated, and proteins were precipitated with cold acetone for 2 h twice. After drying the pellet, proteins were dissolved with 8 M urea and denatured for 1 h at 37 °C with shaking. Proteins were reduced with 10 mM DTT in 50 mM ammonium bicarbonate (ABC) for 1 h. DTT solution was subsequently removed, and proteins were incubated in 40 mM iodoacetamide in 100 mM ABC for 1 h with shaking in the dark. The sample solution was diluted with 50 mM ABC until reaching 1 M urea, after which 1 M CaCl_2_ was added (final concentration, 1 mM). Trypsin (10 µL of 20 ng/µL) was added to 5 µg protein samples, and digestion was completed overnight with shaking at 37 °C. After overnight digestion, excess trypsin solution was removed from each sample, and samples were incubated with streptavidin-coated magnetic beads (Invitrogen, #11205D) for 1 h with gentle rotation. Streptavidin beads were washed twice with 2 M urea in 50 mM ABC. Biotinylated proteins were eluted from magnetic streptavidin beads with elution buffer (acetonitrile/TFA/formic acid/H_2_O = 80:0.2:0.1:19.7, v/v/v) for 5 min at 60 °C five times. Combined extracts were dried to completeness in a vacuum concentrator, reconstituted in 0.1% formic acid/3% acetonitrile, and analyzed as previously described^21^.

### Bioinformatics meta-analysis

Comparison of expression patterns for LONP1 and ClpP was performed using cancer databases from TCGA (http://tcga-data.nci.nih.gov/tcga/) and the online University of California Santa Cruz Xena browser (http://xena.ucsc.deu/). Kaplan–Meier analysis was based on the TCGA PRAD database, and a log-rank (Mantel–Cox) test was performed to evaluate statistical significance.

### Quantification and statistical analysis

All statistical analyses were performed using GraphPad Prism 7. Data are shown as the mean ± standard deviation (SD) of two or three independent experiments. A *p*-value of ≤0.05 was considered statistically significant. Pearson’s correlation coefficients (*r*) were used to calculate the correlation between LONP1 and ClpP. Survival was assessed using the Kaplan–Meier method and log-rank test.

## Results

### LONP1 and ClpP are highly co-expressed in multiple human cancers

**Fig. 1:**
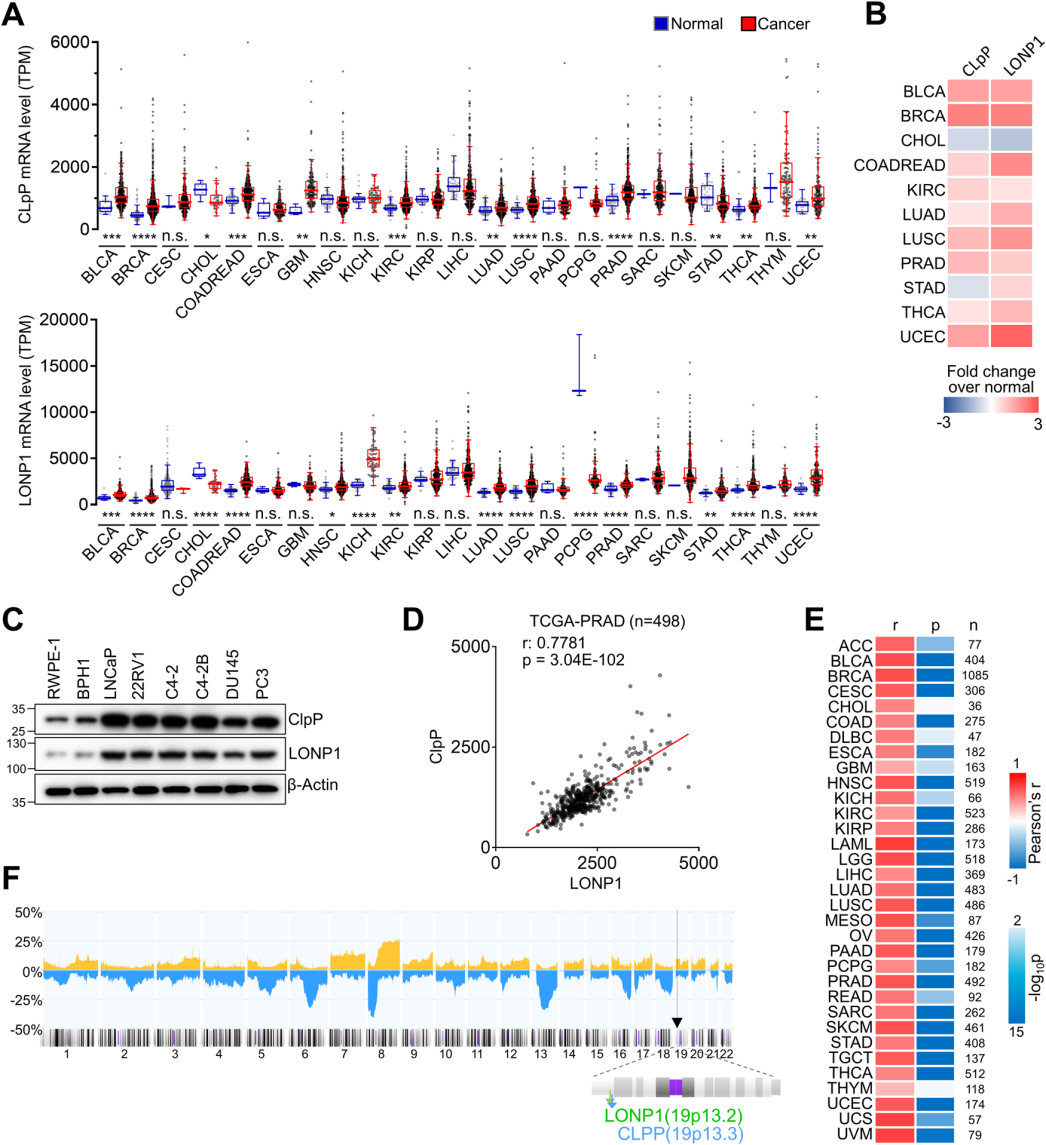
Co-expression of ClpP and LONP1 in human cancers. **A** mRNA expression of ClpP (upper) and LONP1 (bottom) were compared between multiple tumor types and paired normal tissue from the TCGA database. Data are shown as a box and whisker plot using the Tukey method. Student’s t-tests were used to evaluate statistical significance. *p < 0.05, **p < 0.01, ***p < 0.001, ****p < 0.0001, n.s., not significant. **B** Heatmap showing summary mRNA expression profiles of ClpP and LONP1 in multiple tumor types with statistical significance (*p* < 0.05) (cancer vs. normal, red: upregulation; blue: downregulation). **C** Protein levels of ClpP and LONP1 in cell lines analyzed by western blotting. **D** Co-expression analysis of ClpP and LONP1. mRNA expression values from RNA Seq FPKM data from the TCGA PCa database (*n* = 498). Pearson correlation coefficients (*r*) and *p*-values (p) are presented. **E** Co-expression analysis of ClpP and LONP1 in multiple cancer types with statistical significance (*p* < 0.05) from the TCGA database. Pearson correlation coefficients (*r*), *p*-values (*p*), and number of patients (*n*) are presented. **F** Frequency of genomic gains/amplifications (orange, up) and deletions (blue, down) across all chromosomes from 1,323 prostate cancer samples shown in a Progenetix histoplot. The genomic locus of LONP1 and ClpP genes are marked by arrowheads.

To investigate relationships between the two proteases, we first analyzed patterns of LONP1 and ClpP expression in various cancer types from The Cancer Genome Atlas (TCGA) database. RNA expression of both LONP1 and ClpP were significantly upregulated in a wide range of human cancers, including bladder, breast, colon, kidney, lung, thyroid, uterine, and prostate cancer, compared with normal tissue (Fig. 1A). Interestingly, their expression pattern was markedly similar across cancer types (Fig. 1B). In addition, in the Cancer Cell Line Encyclopedia (CCLE) database containing 1457 cancer cell lines, median ClpP expression was the highest in prostate cancer, and LONP1 expression was also highly elevated in prostate cancer relative to other cancer types (Fig. S1). Given the similar elevated expression patterns of these two mitochondrial proteases in tumor tissue compared with normal tissue, we hypothesized that co-expression of these two proteases is associated with cancer progression. In agreement with mRNA data, LONP1 and ClpP proteins were highly co-expressed in prostate cancer compared with non-transformed prostate cells, including RWPE1 and BPH1 cells (Fig. 1C). Furthermore, LONP1 and ClpP mRNA expression were strongly positively correlated in prostate cancer patient samples as well as most other cancer samples (Fig. 1D, E; Fig. S2), whereas human m-AAA protease AFG3L2 had no correlation with LONP1 and ClpP (Fig. S3). Interestingly, LONP1 and ClpP genes closely localized to chromosomal region 19 (19q13), and the genomic locus of these two genes frequently exhibited gain in prostate cancer patient samples (Fig. 1F). This finding that LONP1 and ClpP are highly co-expressed in various human cancers suggests a functional link between the two mitochondrial proteases in cancer.

### Inhibition of LONP1 and ClpP additively leads to cancer cell death

**Fig. 2:**
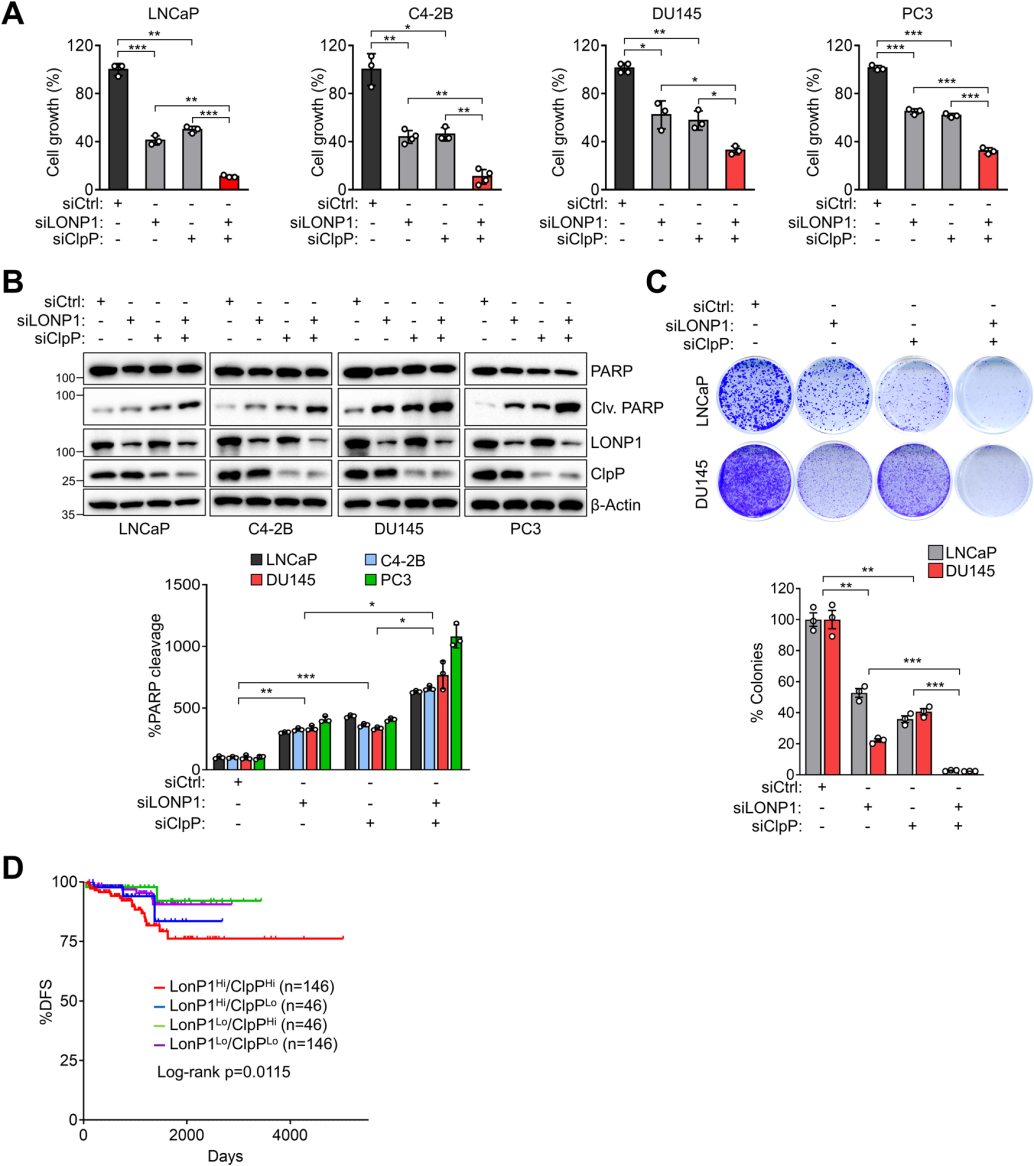
Inhibition of ClpP and LONP1 attenuates cancer cell growth and induces cell death. **A**, **B** Prostate cancer cell lines (LNCaP, C4–2B, DU145, and PC3) were transfected with control non-targeting siRNA (siCtrl) or LONP1 (siLONP1) and/or ClpP-directed siRNA (siClpP) for 72 h. **A** Cell proliferation was measured by direct cell counting. Mean ± SD (*n* = 3). *p < 0.01, **p < 0.001, ***p < 0.0001. **B** Total cell lysates were analyzed by western blotting for full-length and cleaved PARP protein levels as an apoptotic marker. Clv, cleaved. Densitometric quantification of bands in cleaved PARP was normalized with bands in total PARP (bottom). Data from three independent experiments are shown. Mean ± SD (*n* = 3). *p < 0.01, **p < 0.001, ***p < 0.0001. **C** LNCaP and DU145 cells were transfected with siCtrl or siLONP1 and/or siClpP for 48 h and reseeded in 6-well plates. Colony formation was assessed by crystal violet staining after 7 days and quantified (bottom). Data from three independent experiments are shown. Mean ± SD (*n* = 3). **p < 0.001, ***p < 0.0001. **D** Kaplan–Meier recurrence-free survival of patients from the TCGA prostate cancer database (*n* = 471).

Inhibition of LONP1 and ClpP separately reduces cancer growth and viability^22,14^. To assess whether co-expression of these two proteases plays a role in cancer, we examined whether LONP1 and ClpP downregulation jointly impact the proliferation of prostate cancer cells. Separate knockdown of LONP1 or ClpP genes with small interfering RNA (siRNA) reduced the growth of prostate cancer cells, including LNCaP, C4-2B, DU145, and PC3 cells. However, the simultaneous knockdown of LONP1 and ClpP reduced cell growth more than a single knockdown and caused cell death, as evidenced by increased PARP cleavage (Fig. 2A, B). To confirm these results, we performed the colony formation assay and observed that the simultaneous knockdown of both genes significantly reduced colony formation (Fig. 2C). Conversely, the silencing of LONP1 and ClpP did not affect the growth of the non-cancerous cell lines BPH-1 and HEK-293, which had lower levels of LONP1 and ClpP than those in cancer cells (Fig. S4). Consistently, prostate cancer patients with high levels of both LONP1 and ClpP expression showed significantly worse survival outcomes than those with only high LONP1 or ClpP expression (Fig. 2D). Together, these findings further indicate a link between LONP1 and ClpP function in cancer cell proliferation and viability.

### LONP1 and ClpP maintain mitochondrial functions and cell survival under stress conditions

**Fig. 3:**
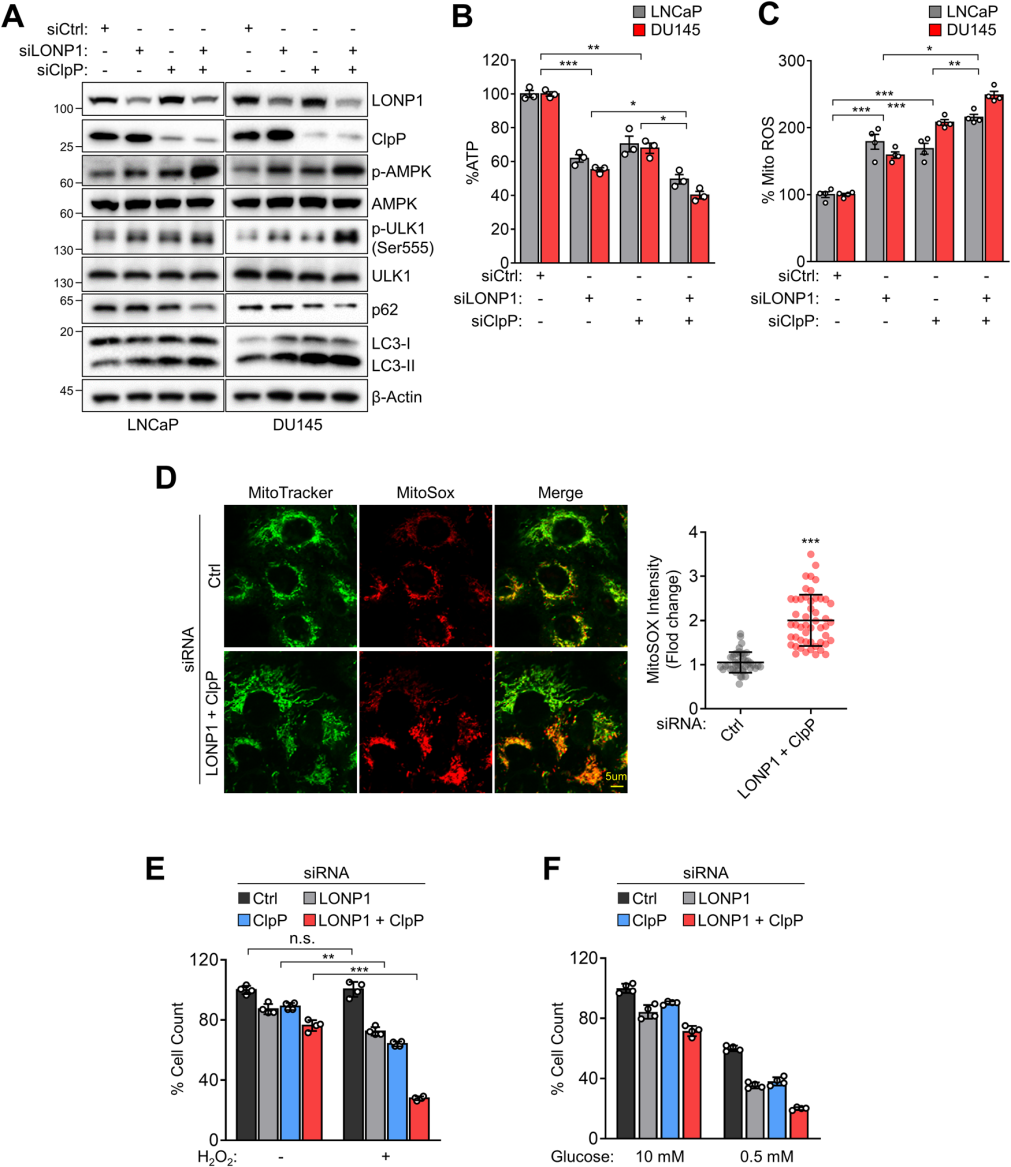
ClpP and LONP1 protect mitochondria functions required for cell survival under stress conditions. **A**–**F** LNCaP and DU145 cells were transfected with control non-targeting siRNA (siCtrl) or LONP1 (siLONP1) and/or ClpP-directed siRNA (siClpP) for 72 h. **A** Total cell lysates were analyzed by western blotting for phosphorylation of AMPK (Thr172) and ULK1 (Ser555), p62 and LC3-I/II. p, phosphorylated. **B** ATP production. **C** Mitochondrial ROS levels. Data representative of three independent experiments are shown. Mean ± SD (*n* = 3). *p < 0.05, **p < 0.01, ***p < 0.001. **D** Representative images of MitoSOX Red (for mitochondrial superoxide) and MitoTracker Green (for mitochondrial content) fluorescence. Scale bar = 5 µm. Quantitative analysis of fluorescence intensity is shown in the right panel. Integrated pixel intensities from MitoSOX Red signals were normalized by the area occupied by MitoTracker Green. Whole images and 40 cells per group were used for calculations. Data are shown as mean ± SD of individual cells. ***p < 0.001. **E**, **F** DU145 cells were transfected with siCtrl or siLONP1 and/or siClpP for 48 h. Cells were incubated in (**E**) H_2_O_2_ (100 μM) or (**F**) low glucose (0.5 mM) for 24 h. Cell proliferation was measured by direct cell counting. Mean ± SD (*n* = 3). **p < 0.001, ***p < 0.0001, n.s., not significant.

We next investigated the role of LONP1 and ClpP co-expression in the regulation of mitochondrial function. Knockdown of LONP1 or ClpP in LNCaP or DU145 cells led to increased phosphorylation of the energy sensor 5’-adenosine monophosphate-activated protein kinase (AMPK), indicating defective mitochondrial bioenergetics^23,24^ (Fig. 3A, Fig. S5). In turn, autophagy, a downstream pathway of AMPK signaling, was upregulated by LONP1 or ClpP knockdown, as evidenced by the phosphorylation of ULK1, reduction in p62, and LC3-II accumulation. In addition, LONP1 or ClpP knockdown increased the protein or mRNA expression of mitochondrial-UPR (mito-UPR) genes, including CHOP, Hsp60, ATF4, and ATF6 (Fig. S6). However, the double knockdown of LONP1 and ClpP increased AMPK phosphorylation, mito-UPR, and autophagy more than single knockdowns (Fig. 3A, Figs. S5, S6). Consistently, the depletion of LONP1 or ClpP reduced ATP production (Fig. 3B) and increased mitochondrial ROS production (Fig. 3C, D), whereas normal cells were not affected (Fig. S4D, E). LONP1 and ClpP silencing did not affect the total mitochondrial content (Fig. S7). In addition, AMPK phosphorylation, autophagy, and mito-UPR pathway activity, and ROS production were further increased after the simultaneous silencing of both genes. Considering the pro-survival roles of LONP1 and ClpP, we next evaluated LONP1- and ClpP-mediated mitochondrial protease requirements for cancer cell adaptation to metabolic stressors, such as starvation and oxidative stress. Depletion of LONP1 or ClpP increased cell sensitivity to metabolic stress induced by oxidative stress (H_2_O_2_; Fig. 3E) or starvation (low glucose; Fig. 3F), and this cell sensitivity was further increased by simultaneous knockdown of both genes. These findings suggest that LONP1 and ClpP cooperatively modulate mitochondrial function in cancer cell survival.

### LONP1 and ClpP regulate mitochondrial protein folding and proteostasis

**Fig. 4:**
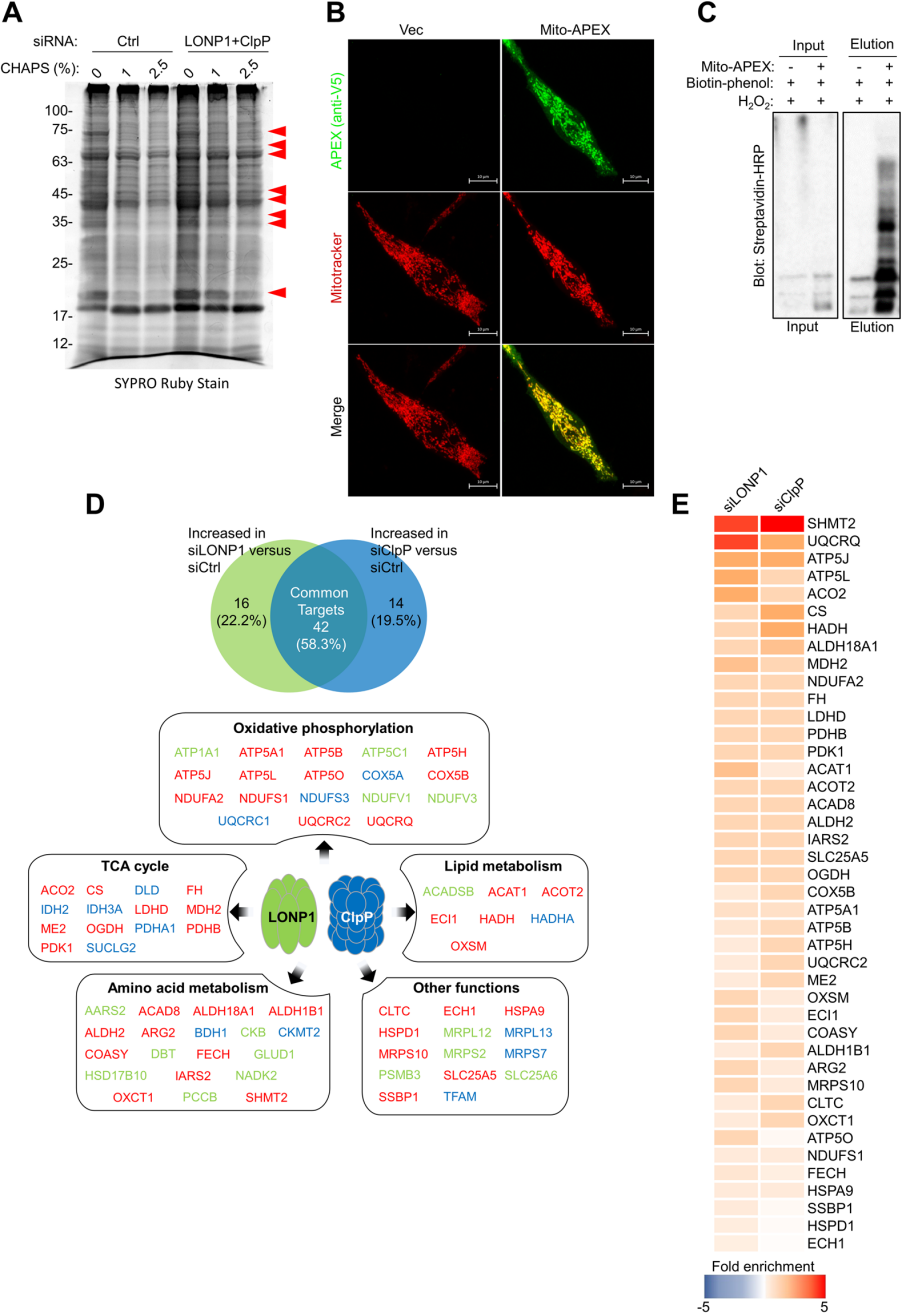
Mitochondrial protein quality control by ClpP and LONP1. **A** Mitochondrial proteins from LNCaP cells treated with control non-targeting siRNA (siCtrl) or LONP1 (siLONP1) and ClpP-directed siRNA (siClpP) for 48 h were incubated with increasing concentrations of detergent (CHAPS), separated by SDS gel electrophoresis, and visualized by SYPRO Ruby Stain. Aggregated and misfolded mitochondrial proteins are marked with arrowheads. **B** Representative confocal images of mitochondrial morphology in LNCaP cells expressing mito-APEX. Twenty-four hours after transfection, cells were fixed and stained with anti-V5 to detect mito-APEX expression. Mitotracker Deep Red staining was also used to visualize mitochondria. Scale bar=10 μm. **C** LNCaP cells were transiently transfected with mito-APEX. Twenty-four hours later, cells were incubated with the APEX substrate biotin-phenol and H_2_O_2_ for 1 min. Following cell lysis, biotinylated species were enriched by streptavidin pulldown. Western blot analysis of biotinylated mitochondrial proteins before (input) and after (elution) streptavidin bead enrichment. **D** APEX-based mitochondrial proteome analysis quantified by liquid chromatography-mass spectrometry for identification of direct target proteins for LONP1 or ClpP. Levels of 72 proteins were increased with a *p* < 0.05 and fold change ≥1.5 versus control siRNA in two independent experiments (*n* = 3). Upper, Venn diagram showing the number of mitochondrial proteins upregulated by silencing of LONP1 or ClpP. Bottom, target proteins categorized according to Gene Ontology enrichment analysis (DAVID). Red, common targets of LONP1 and ClpP; green, targets of LONP1; blue, targets of ClpP. **E** Fold increase in mitochondrial proteins with *p* < 0.05 that were common targets of ClpP and LONP1. Fold enrichment was determined relative to LNCaP cells transfected control siRNA. Data from two independent experiments are shown.

The function of mitochondrial proteases is determined by maintaining protein quality through the specific proteolysis of damaged proteins. To investigate the molecular mechanisms by which LONP1 and ClpP regulate mitochondrial function, we characterized LONP1- and ClpP-specific substrates. We first examined the impact of LONP1 and ClpP depletion on mitochondrial protein homeostasis. siRNA silencing of LONP1 and ClpP in LNCaP cells resulted in the accumulation of detergent-insoluble proteins, indicating accumulation of aggregated and misfolded proteins (Fig. 4A). Based on this finding, we identified potential substrates of proteases by measuring levels of upregulated proteins in the detergent-insoluble fraction after LONP1 and ClpP depletion with comprehensive proteomic analysis^19^. We also applied the mitochondrial matrix-targeted ascorbate peroxidase (mito-APEX) labeling method to probe the spatial mitochondrial matrix proteome (Fig. S8)^25,21^. We transiently expressed mito-APEX in LNCaP cells and confirmed that mito-APEX was specifically expressed in mitochondria and that endogenous proteins were biotinylated by mito-APEX (Fig. 4B and C). Mitochondria matrix proteins were purified using streptavidin beads and quantified by mass spectrometry analysis. In total, all 144 mitochondrial matrix proteins were detected. Of these, 72 proteins showed increased levels (≥1.5-fold change) in spectral counts (p < 0.05) following knockdown of LONP1 and ClpP (Fig. 4D), indicating a requirement of LONP1 and ClpP for specific proteolysis. Subsequently, 42 proteins (58.3% of total) were commonly upregulated by silencing of LONP1 or ClpP, indicating that LONP1 and ClpP share substrate proteins (Fig. 4D and E). Among the substrates displaying large increases were components of oxidative phosphorylation, the TCA cycle, and lipid or amino acid metabolism, and the substrate with the most robust increase was serine hydroxymethyltransferase-2 (SHMT2), which is a key regulator of serine metabolism in mitochondria (Fig. 4E). These results suggest that ClpP and LONP1 selectively but cooperatively modulate mitochondrial proteostasis.

### SHMT2 is a common substrate of ClpP and LONP1 proteases

**Fig. 5:**
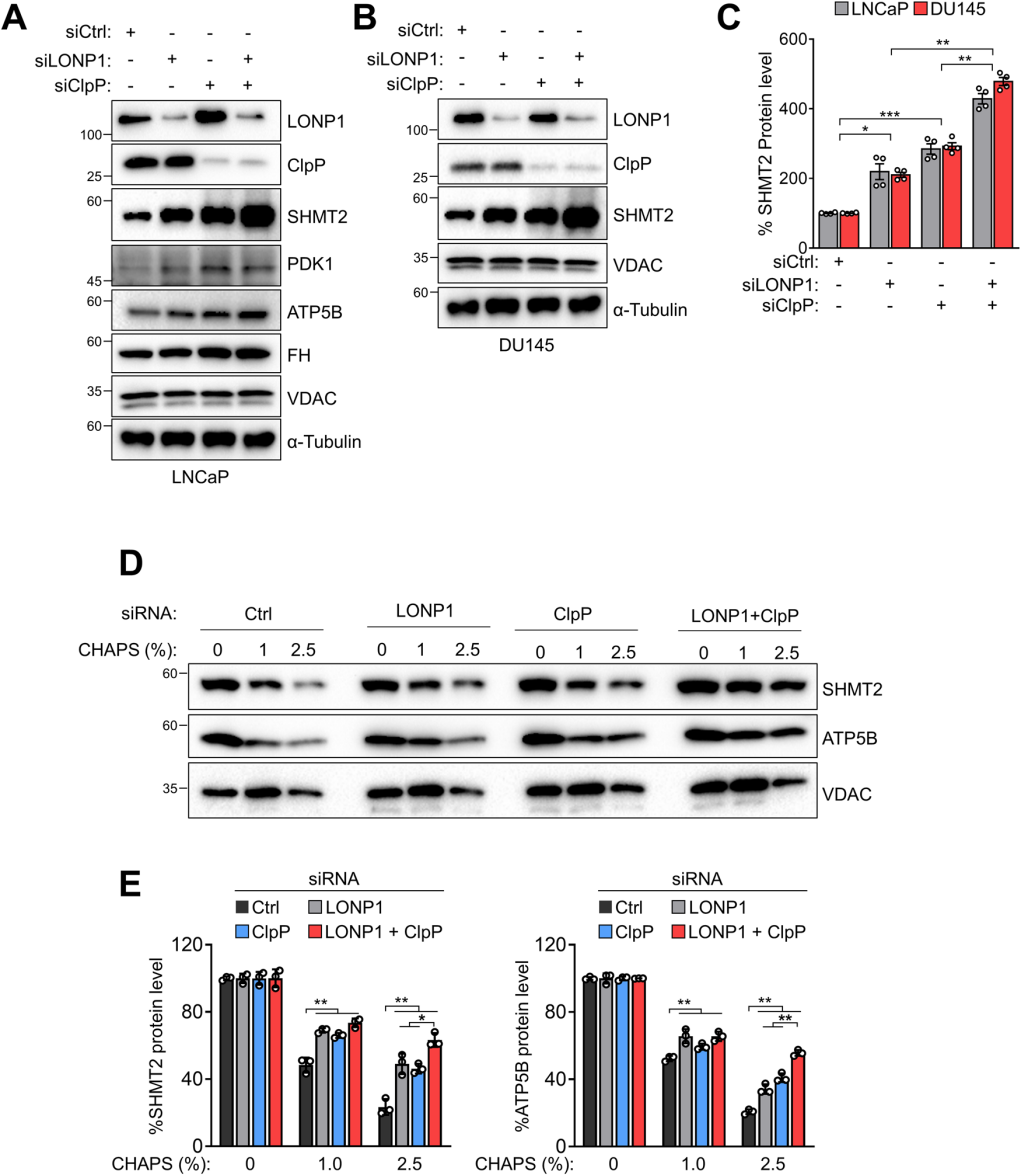
ClpP and LONP1 cooperatively modulate SHMT2 folding. **A**–**C** LNCaP and DU145 cells were transfected with control non-targeting siRNA (siCtrl) or LONP1 (siLONP1) and/or ClpP-directed siRNA (siClpP) for 72 h. **A**, **B** Total cell lysates were analyzed by western blotting. **C** Densitometric quantification of SHMT2/α-tubulin bands. Mean ± SD (*n* = 3). *p < 0.01, **p < 0.001, ***p < 0.0001. **D**, **E** Mitochondria from DU145 cells transfected with siCtrl or siLONP1 and/or siClpP for 72 h were mixed with increasing concentrations of CHAPS, and insoluble fractions were analyzed by western blotting (**D**). **E** Densitometric quantification of bands in SHMT2 or ATP5B were normalized with bands in their VDAC. Data from three independent experiments are shown. Mean ± SD (*n* = 3). *p < 0.01, **p < 0.001.

To confirm our proteomic results, we analyzed protein levels of substrate targets for LONP1 and ClpP by western blotting with specific antibodies against several proteins including SHMT2, pyruvate dehydrogenase kinase 1 (PDK1), ATP synthase subunit β (ATP5B), and fumarate hydratase (FH) (Fig. 5A and Fig. S9). The silencing of LONP1 or ClpP increased these protein levels, particularly SHMT2, which was significantly increased following the silencing of both genes (Fig. 5A). An increase in the SHMT2 level by LONP1 and ClpP knockdown was also observed in DU145 cells, whereas the expression level of cytosolic SHMT1 was not affected (Fig. 5B, C; Fig. S10). Next, we examined the folding status of substrate proteins in the absence of LONP1 and ClpP. We found that knockdown of LONP1 or ClpP selectively induced accumulation of misfolded or aggregated SHMT2 and ATP5B at different CHAPS concentrations (Fig. 5D, E) under the same conditions. These results suggest that LONP1 and ClpP selectively and cooperatively regulate SHMT2 protein quality by degrading unfolded SHMT2 proteins.

### LONP1- and ClpP-directed SHMT2 protein homeostasis is required for cancer cell survival

**Fig. 6:**
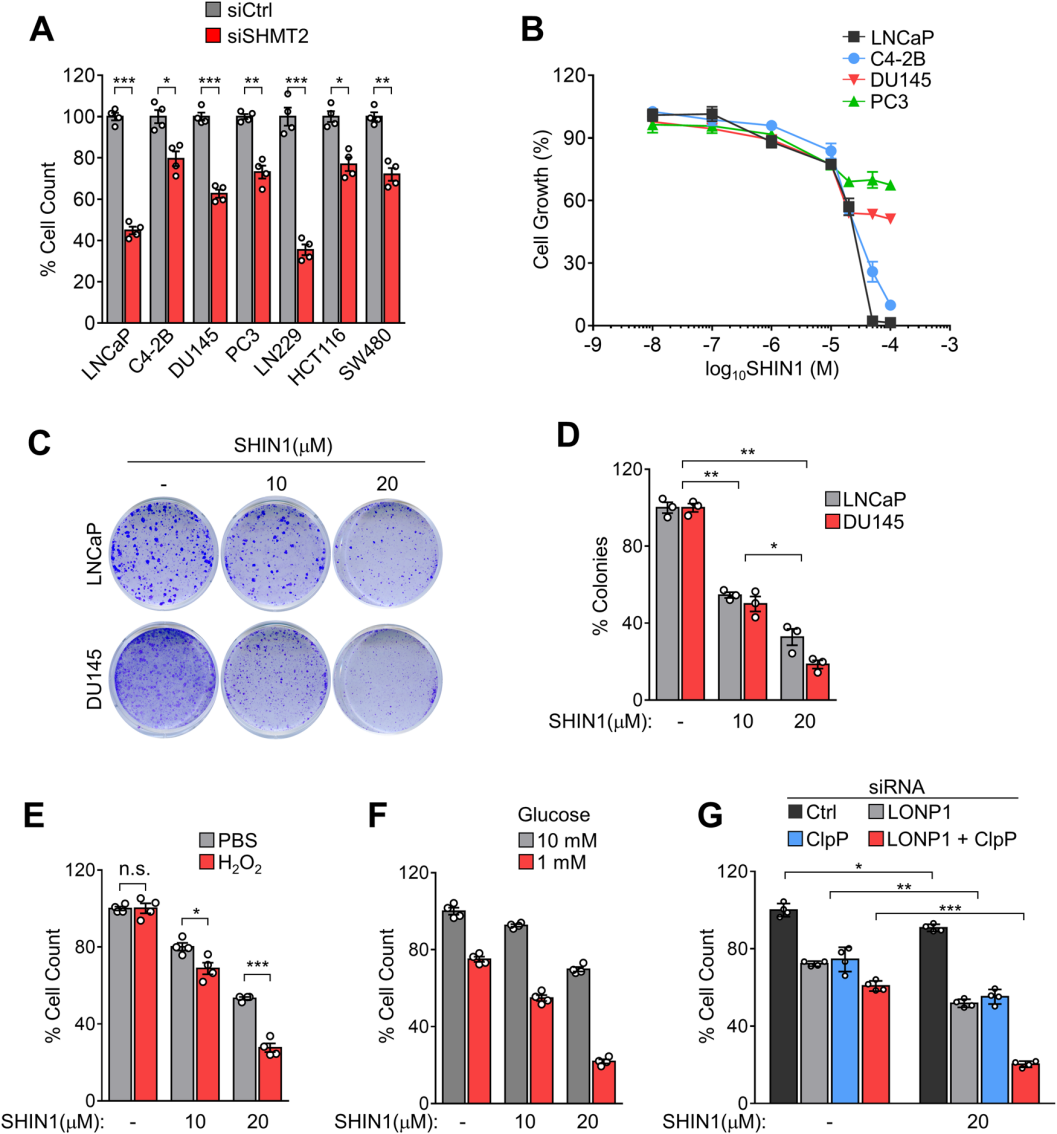
SHMT2 inhibition reduces cancer cell proliferation and survival under stress conditions. **A** Cell lines were transfected with control non-targeting siRNA (siCtrl) or SHMT2-directed siRNA (siSHMT2) for 48 h. Cell proliferation was measured by direct cell counting. Mean ± SD (*n* = 4). *p < 0.01, **p < 0.001, ***p < 0.0001. **B** Prostate cancer cell lines (LNCaP, C4–2B, DU145, and PC3) were treated with increasing concentrations (0–100 μM) of SHIN1 for 48 h, Cell proliferation was measured by direct cell counting. Mean ± SD (*n* = 3). **C**, **D** LNCaP and DU145 cells were plated and treated with or without SHIN1 (10 or 20 μM) for colony formation assay. Colonies were analyzed by crystal violet staining after 10 days and quantified (**D**). Mean ± SD (*n* = 3). *p < 0.01, **p < 0.001. **E**, **F** DU145 cells were treated with or without SHIN1 (10 or 20 μM) for 24 h. Cells were incubated in (**E**) H_2_O_2_ (50 μM) or (**F**) low glucose (1 mM) for 24 h. Cell proliferation was measured by direct cell counting. Mean ± SD (*n* = 4). *p = 0.0210, ***p < 0.0001. **G** DU145 cells were transfected with control non-targeting siRNA (siCtrl) or LONP1 (siLONP1) and/or ClpP-directed siRNA (siClpP) for 48 h. Cells were incubated with SHIN1 (20 μM) for 24 h. Cell proliferation was measured by direct cell counting. Mean ± SD (*n* = 4). *p = 0.0032, **p < 0.001, ***p < 0.0001.

Serine is a central hub of cancer metabolism that sustains cell growth and proliferation^26^. Also, upregulation of SHMT2 and its association with tumor progression is reported for several types of cancer^27,28,29^. Therefore, we examined whether SHMT2 inhibition affects the proliferation and survival of cancer cells. Knockdown of SHMT2 attenuated the growth of prostate cancer cells (LNCaP, C4-2B, DU145, and PC3 cells) as well as various cancer cell lines including glioblastoma line LN229 and colon cancer cell lines HCT116 and SW480 (Fig. 6A) following gene silencing by siRNA (Fig. S11). In addition, treatment with a chemical inhibitor of SHMT2 (SHIN1) inhibited the growth of LNCaP and C4-2B cells in a dose-dependent manner and partially reduced the growth of DU145 and PC3 cells (Fig. 6B). Interestingly, SHIN1 significantly inhibited colony formation, a sign of cancer growth, in LNCaP and DU145 cells (Fig. 6C, D). Consistent with an increased sensitivity to cellular stress after LONP1 and ClpP knockdown, we observed that inhibition of SHMT2 enhanced cancer cell sensitivity to stressors such as oxidative stress (H_2_O_2_, Fig. 6E) or starvation (low glucose, Fig. 6F). Furthermore, the reduction of cell growth by simultaneous silencing of LONP1 and ClpP was significantly more pronounced following SHIN1 treatment compared with that in separate silencing experiments, whereas control siRNA-transfected cells showed only a marginal effect following SHIN1 treatment (Fig. 6G) These results demonstrate that SHMT2 is a key substrate of LONP1 and ClpP in cancer growth and resistance to cellular stress.

## Discussion

To survive inhospitable tumor environments, tumor cells activate stress adaptation pathways to buffer stress-induced cytotoxicity. In turn, successful adaptation to diverse cytotoxic stress provides cancer cells with survival and proliferation advantages, leading to therapeutic resistance, dormancy, and metastasis^4^. In these stress adaptation processes, proteolytic removal of misfolded, denatured, or oxidized proteins is important for the integrity of subcellular organelles, especially mitochondria, which possess their own proteolytic systems that are evolutionarily conserved from bacterial cells^30^. However, the detailed molecular mechanisms by which mitochondrial proteases coordinate their functions to maintain proteostasis in cancer remain largely unknown. In this study, we found evidence of functional crosstalk between LONP1 and ClpP within the mitochondrial protein quality control pathway required for cancer cell survival. Depletion of LONP1 and ClpP led to the accumulation of misfolded proteins and inhibited cell growth under environmental stress conditions. Notably, as both proteases share mitochondrial substrate targets, the absence of both genes additively inhibited cell growth, disrupted mitochondrial function, and increased oxidative stress, leading to cancer cell death. Furthermore, co-expression of both proteins at high levels in multiple tumor types was associated with shortened patient survival, strongly supporting a functional link between LONP1 and ClpP in human cancer.

Mitochondria have developed several stress response mechanisms to maintain their homeostasis^31–33,12^. In addition to selective removal of damaged mitochondria through mitophagy, first-line defense mechanisms against mitochondrial damage occur on a molecular level via proteolytic machineries consisting of chaperones and proteases. Apart from the stabilizing action of molecular chaperones, the major defense mechanism against the accumulation of damaged proteins is their specific removal by proteolysis. To date, at least 15 mitochondrial proteases have been identified, including LONP1 and ClpP, which are soluble AAA + mitochondrial proteases in the mitochondrial matrix that contribute to the maintenance of proteostasis^18,30^. However, the specific substrates of each protease and their functions are still largely unknown. There is increasing evidence that LONP1 and ClpP are involved in cancer. For instance, the silencing of LONP1 causes mitochondrial metabolic dysfunction, cellular senescence, and reduced tumor formation via remodeling of the oxidative phosphorylation complex, whereas overexpression of LONP1 promotes tumorigenesis^14^. In addition, cancer cells exposed to stress stimuli, including hypoxia, exhibit increased LONP1 expression associated with phosphorylation by the Akt pathway, which enhances oxidative metabolism and tumor cell metastatic competence^34^. Dysregulated mitochondrial respiration capacity by ClpP inhibition results in activation of cellular stress, reduced cell proliferation, and impaired metastatic dissemination^15^. Inhibiting ClpP in leukemic cells leads to abnormal protein accumulation and causes cell death^22^. Also, hyperactivation of ClpP activity induces abnormal mitochondrial proteolysis and impairs mitochondrial function, leading to cancer cell death^18^, suggesting that tight regulation of protease activity is important for cancer and pointing toward mitochondrial protease activity as a promising therapeutic target for cancer.

Despite functional similarities between LONP1 and ClpP in cancer, their regulatory mechanisms are largely unknown. Here, we demonstrated that LONP1 and ClpP work together to maintain mitochondrial proteostasis in cancer. LONP1 and ClpP genes closely localized to chromosomal region 19 and were co-expressed at high levels in most human cancers. We revealed that the two proteases share numerous target substrates that are crucial components of mitochondrial functions, including oxidative phosphorylation, the TCA cycle, and amino acid and lipid metabolism. We found that LONP1 and ClpP cooperate to maintain protein quality and the activity of target substrates, which may support cancer cell proliferation and survival from cellular stress. Accordingly, inhibition of both LONP1 and ClpP potently reduced cancer cell viability with concomitantly induced mitochondrial bioenergetics dysfunction as evidenced by phosphorylation of AMPK and activation of autophagy. Furthermore, inhibition of both genes additively reduced cell growth and survival under harsh environmental conditions such as oxidative stress and nutrient deprivation. As an adaptive stress response is associated with aggressive tumor activity, including metastasis and drug resistance, co-targeting both proteases could be an effective therapeutic strategy for advanced cancer.

SHMT2 is the enzyme that converts serine to glycine in one-carbon metabolism, which provides essential substrates for nucleic acid and protein metabolism^35,36^. SHMT2 is highly upregulated in diverse cancer types, correlated with poor outcomes, and may support tumor aggressiveness by enhancing mitochondrial functions, including redox balance and nucleotide synthesis^27,29,37^. In particular, SHMT2 emerged as a key enzyme for the metabolic adaptation of cancer against stress conditions. SHMT2 is required for mitochondrial respiration by supporting oxygen consumption, and an elevated level of SHMT2 reduces mitochondrial pyruvate metabolism and limits oxygen consumption to adapt to the stress^28,38^. Furthermore, the depletion of SHMT2 impaired mitochondrial functions and showed higher cellular ROS levels under metabolic stress conditions such as hypoxia and serine/glycine starvation^28^. In our study, we found that SHMT2 is a common target substrate of LONP1 and ClpP for cancer cell survival. LONP1 or ClpP inhibition increased unfolding of SHMT2. Consistent with the cellular effects of LONP1 and ClpP inhibition, depletion of SHMT also reduced cancer cell growth and survival under cytotoxic stress conditions. Furthermore, depletion of LONP1 and ClpP increased cell sensitivity to an SHMT2 inhibitor, suggesting that SHMT2 is a key substrate in the LONP1- and ClpP-mediated proteostasis network and that its protein quality control by mitochondrial proteases may promote cancer cell survival and progression.

In summary, our study reveals a functional relationship between two mitochondrial proteases LONP1 and ClpP in maintaining mitochondrial proteostasis in cancer. The coordinated regulation of these two proteases, which are commonly altered in different human cancers, appears to protect mitochondrial functions and thereby promote cancer cell growth and survival from cellular stresses. Our results also suggest that targeting the mitochondrial proteostasis network could be an effective therapeutic strategy for cancer. However, as mitochondrial proteostasis is tightly regulated by the coordination of diverse machineries, further studies are required to fully understand the exact mechanisms that regulate mitochondria quality control and their interconnection among proteostasis modules to develop an effective therapeutic strategy for cancer treatment.

## Supplementary information

Legends to Supplementary Figures

Supplementary Figure 1

Supplementary Figure 2

Supplementary Figure 3

Supplementary Figure 4

Supplementary Figure 5

Supplementary Figure 6

Supplementary Figure 7

Supplementary Figure 8

Supplementary Figure 9

Supplementary Figure 10

Supplementary Figure 11

## Data Availability

All data are contained within the manuscript with the exception of the differential expression analysis of LONP1 and CLpP in tumor/normal tissue and various cancer cell lines from The Cancer Genome Atlas (TCGA) and Cancer Cell Line Encyclopedia (CCLE) databases. Further information and requests for resources and reagents should be directed to and will be fulfilled by the Lead Contact, Young Chan Chae (ychae@unist.ac.kr).
