# Supplementary material for: LONP1 and ClpP cooperatively regulate mitochondrial proteostasis for cancer cell survival
Source: Oncogenesis. 2021 Feb 26;10(2):18. doi: 10.1038/s41389-021-00306-1 (PMC7910295; doi:10.1038/s41389-021-00306-1)
Supplement: Supplementary file 1 — Legends to Supplementary Figures [file 41389_2021_306_MOESM1_ESM.docx]

**Legends to Supplementary Figures**

**Supplementary Figure 1.** **Overexpression of ClpP and LONP1 in cancer cell lines.** ClpP (top) and LONP1 (bottom) mRNA expression across cancer cell lines in the CCLE ordered by median expression. Data are presented as a box and whisker plot using the Turkey method; n=1,054 samples across 24 tumor types.

**Supplementary Figure 2. Co-expression analysis for LONO1 and ClpP in multiple cancer types**. mRNA expression values from RNA Seq FPKM data from the TCGA database. Pearson correlation coefficients (r) are presented.

**Supplementary Figure 3. Coexpression analysis for LONP1 (right) and ClpP (left) versus AFG3L2**. mRNA expression values from RNA Seq FPKM data from the TCGA database. Pearson correlation coefficients (r) are presented. n.s., not significant.

**Supplementary Figure 4. Effect of ClpP and LONP1 silencing on normal cell growth and mitochondria functions. (A)** Protein levels of ClpP and LONP1 in cell lines analyzed by western blotting. **(A**-**D)** Benign prostatic hyperplasia epithelial BPH1 and human embryonic kidney 293 (HEK-293) cells were transfected with control non-targeting siRNA (siCtrl) or LONP1 (siLONP1) and/or ClpP-directed siRNA (siClpP) for 72 hr. **(B)** Total cell lysates were analyzed by western blotting. **(C)** Cell proliferation was measured by direct cell counting. **(D)** ATP production. **(E)** Mitochondrial ROS levels. Data representative of three independent experiments are shown. Mean ± SD (n=3). n.s., not significant.

**Supplementary Figure 5. Inhibition of ClpP and LONP1 induces cell death.** LNCaP and DU145 cells were transfected with control non-targeting siRNA (siCtrl) or LONP1 (siLONP1) and/or ClpP-directed siRNA (siClpP) for 72 hr. Total cell lysates were analyzed by western blotting. Densitometric quantification of relative changes in protein expression normalized to their total protein. **(A)** p-AMPK/AMPK, **(B)** p-ULK1/ULK1, **(C)** p62/β-actin, **(D)** LC3-II/β-actin. Data from three independent experiments are shown. Mean ± SD (n=3). *p<0.01, **p<0.001.

**Supplementary Figure 6. Inhibition of ClpP and LONP1 induces mitochondrial stress response.** DU145 cells were transfected with control non-targeting siRNA (siCtrl) or LONP1 (siLONP1) and/or ClpP-directed siRNA (siClpP) for 72 hr. Total cell lysates were analyzed by western blotting. Total RNA was isolated, and the indicated mRNA levels were measured by qRT-PCR. Data from three independent experiments are shown. Mean ± SD (n=3). *p<0.01, **p<0.001.

**Supplementary Figure 7.** **Analysis of mitochondrial content.** Relative mtDNA amount measured by qPCR. Data from three independent experiments are shown. Mean ± SD (n=3). n.s., not significant.

**Supplementary Figure 8.** **Labeling scheme for mitochondrial matrix proteins misfolded by LON1 and ClpP knockdown.** Mito-APEX genetically target proteins in mitochondrial matrix. Labeling was initiated by biotinylation and pull-downed with streptavidin-coated beads, eluted, separated on a gel, and analyzed by MS for identification of target protein.

**Supplementary Figure 9.** **ClpP and LONP1 cooperatively modulate protein folding.** LNCaP cells were transfected with control non-targeting siRNA (siCtrl) or LONP1 (siLONP1) and/or ClpP-directed siRNA (siClpP) for 72 hr. Total cell lysates were analyzed by western blotting. Densitometric quantification of PDK1, ATP5B, and FH normalized to α-tubulin. Mean ± SD (n = 3). *p<0.01, **p<0.001.

**Supplementary Figure 10.** **ClpP and LONP1 cooperatively modulate mitochondrial SHMT2 folding.** LNCaP cells were transfected with control non-targeting siRNA (siCtrl) or LONP1 (siLONP1) and/or ClpP-directed siRNA (siClpP) for 72 hr, fractionated into cytosolic (Cyto) and mitochondrial (Mito) extracts and analyzed by western blotting.

**Supplementary Figure 11.** **Knockdown of SHMT2 by siRNA.** Indicated cell lines were transfected with control non-targeting siRNA (siCtrl) or SHMT2-directed siRNA (siSHMT2) for 48 hr. **(A)** Total cell lysates were analyzed by western blotting. **(B)** Total RNA was isolated, and the indicated mRNA levels were measured by qRT-PCR. Data from three independent experiments are shown. Mean ± SD (n=3). ***p<0.0001.
