# Supplementary figures and images for: LONP1 and ClpP cooperatively regulate mitochondrial proteostasis for cancer cell survival
Source: Oncogenesis. 2021 Feb 26;10(2):18. doi: 10.1038/s41389-021-00306-1 (PMC7910295; doi:10.1038/s41389-021-00306-1)

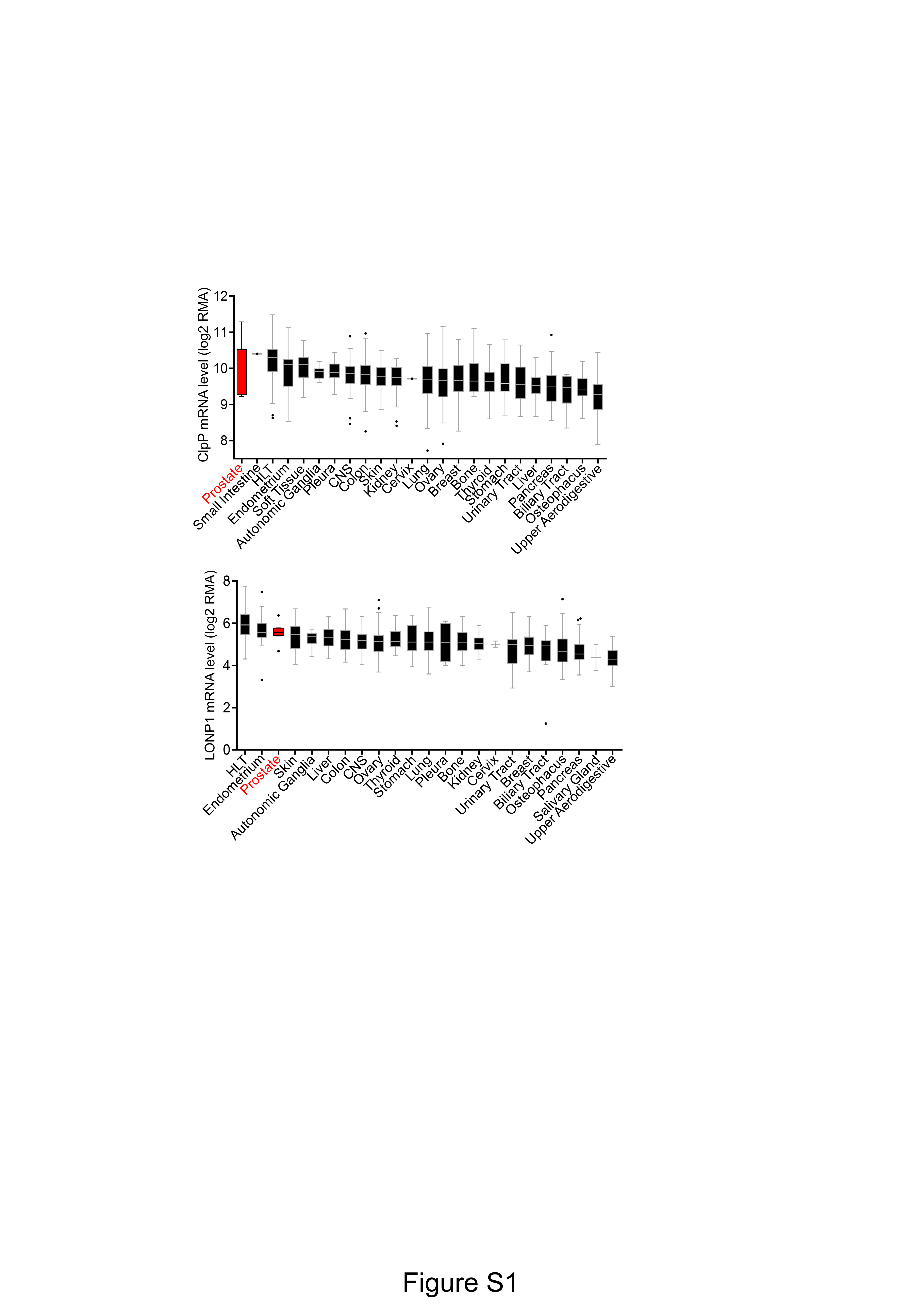

Supplement: Supplementary file 2 — Supplementary Figure 1 [file 41389_2021_306_MOESM2_ESM.tif]

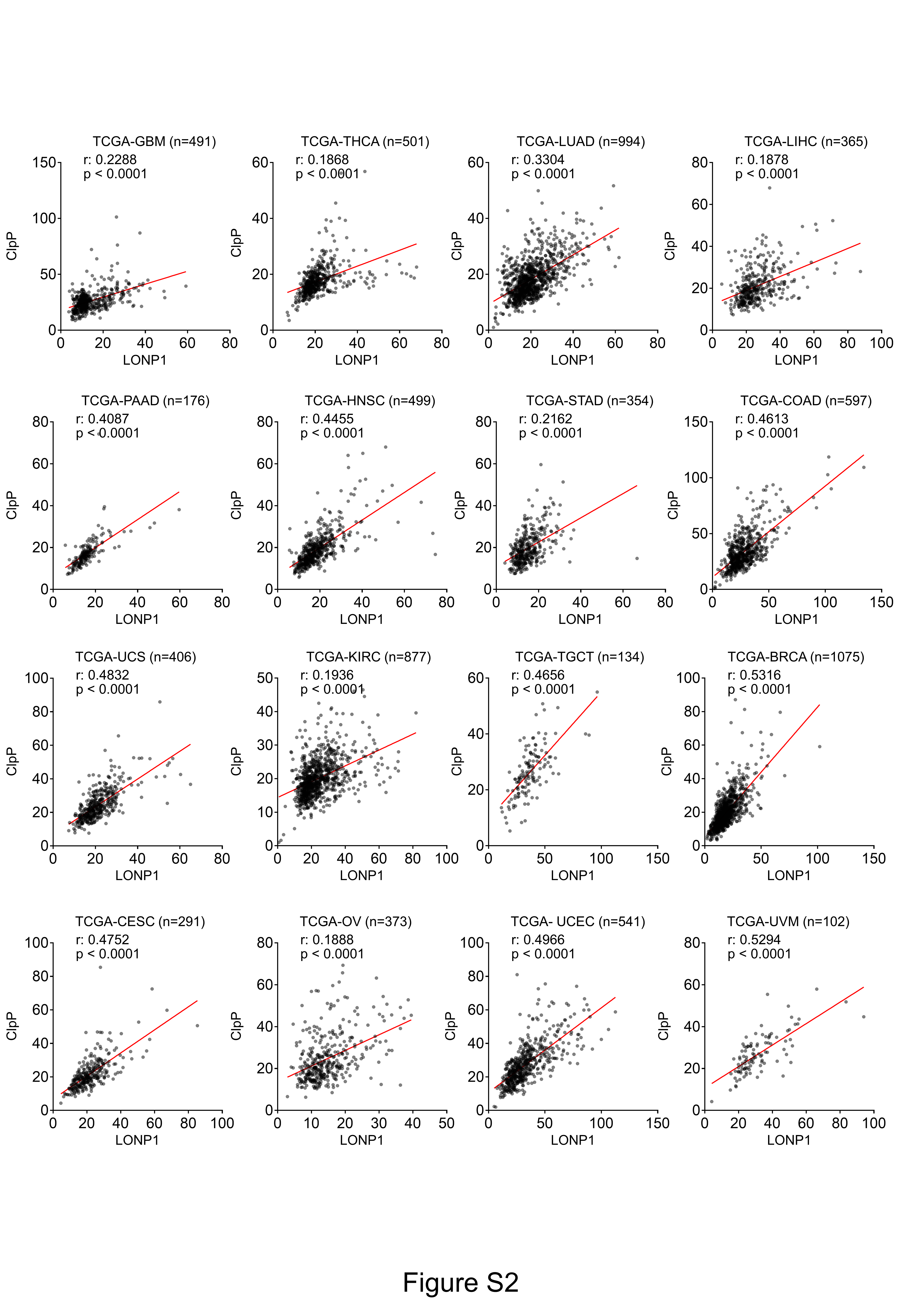

Supplement: Supplementary file 3 — Supplementary Figure 2 [file 41389_2021_306_MOESM3_ESM.tif]

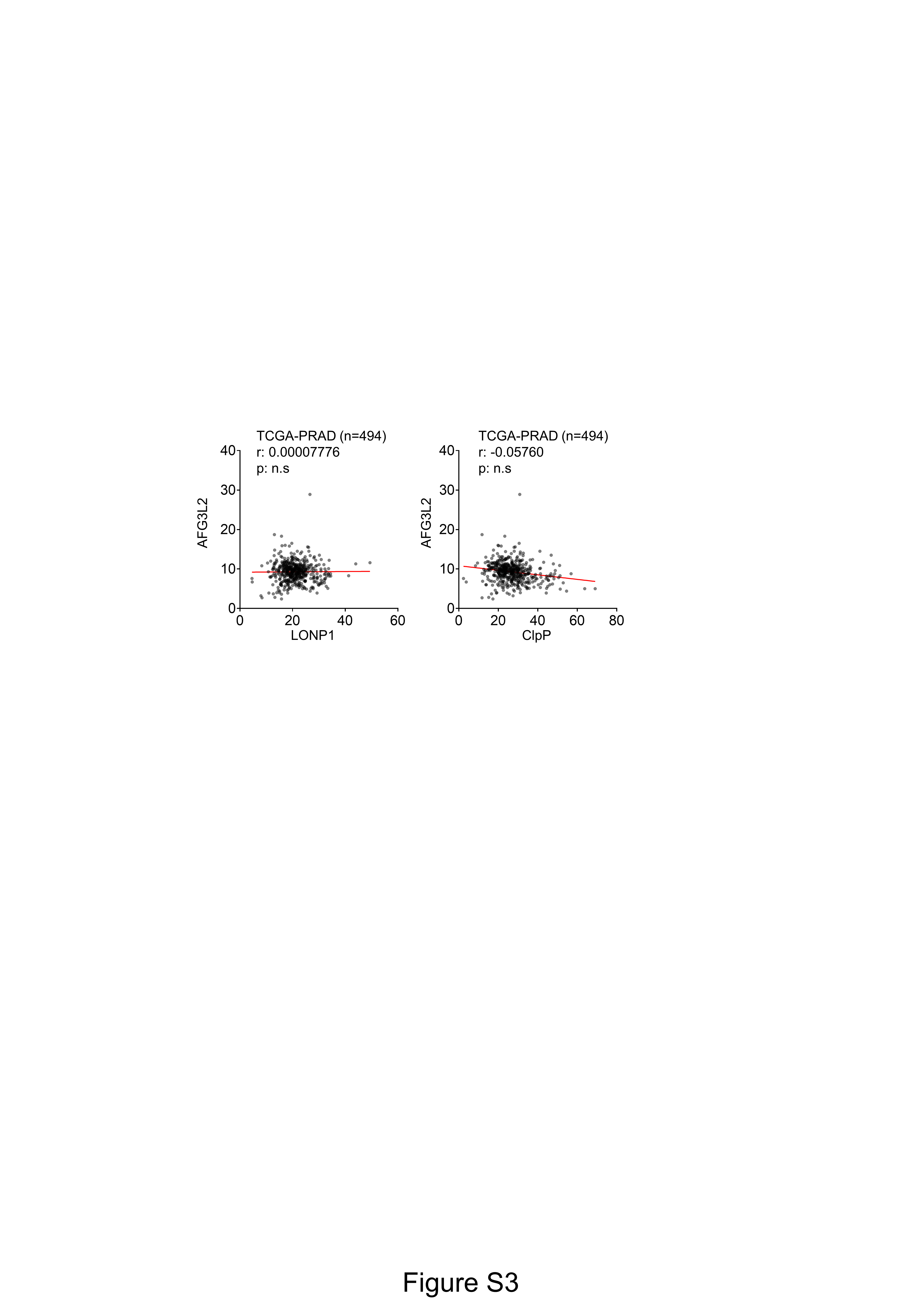

Supplement: Supplementary file 4 — Supplementary Figure 3 [file 41389_2021_306_MOESM4_ESM.tif]

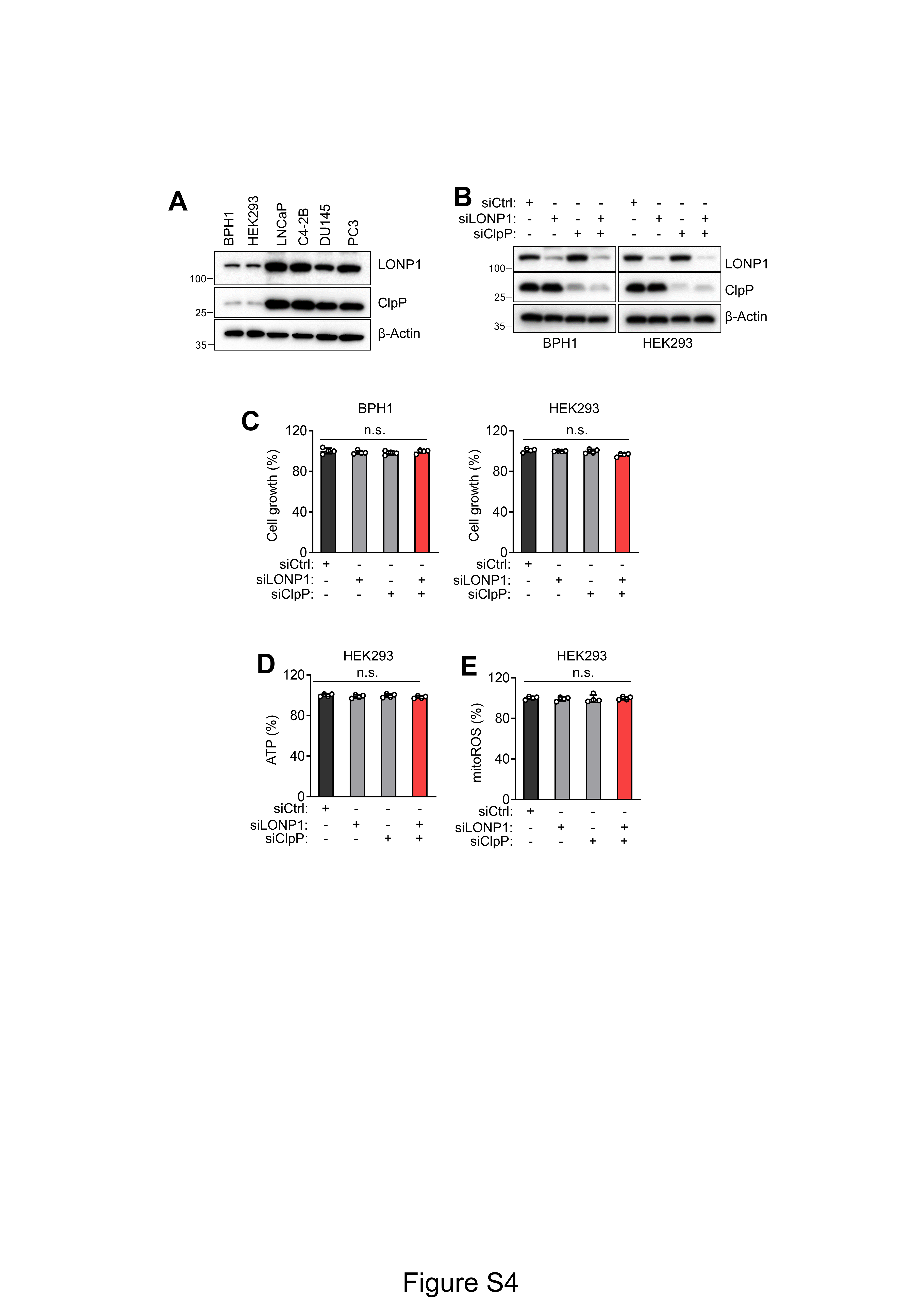

Supplement: Supplementary file 5 — Supplementary Figure 4 [file 41389_2021_306_MOESM5_ESM.tif]

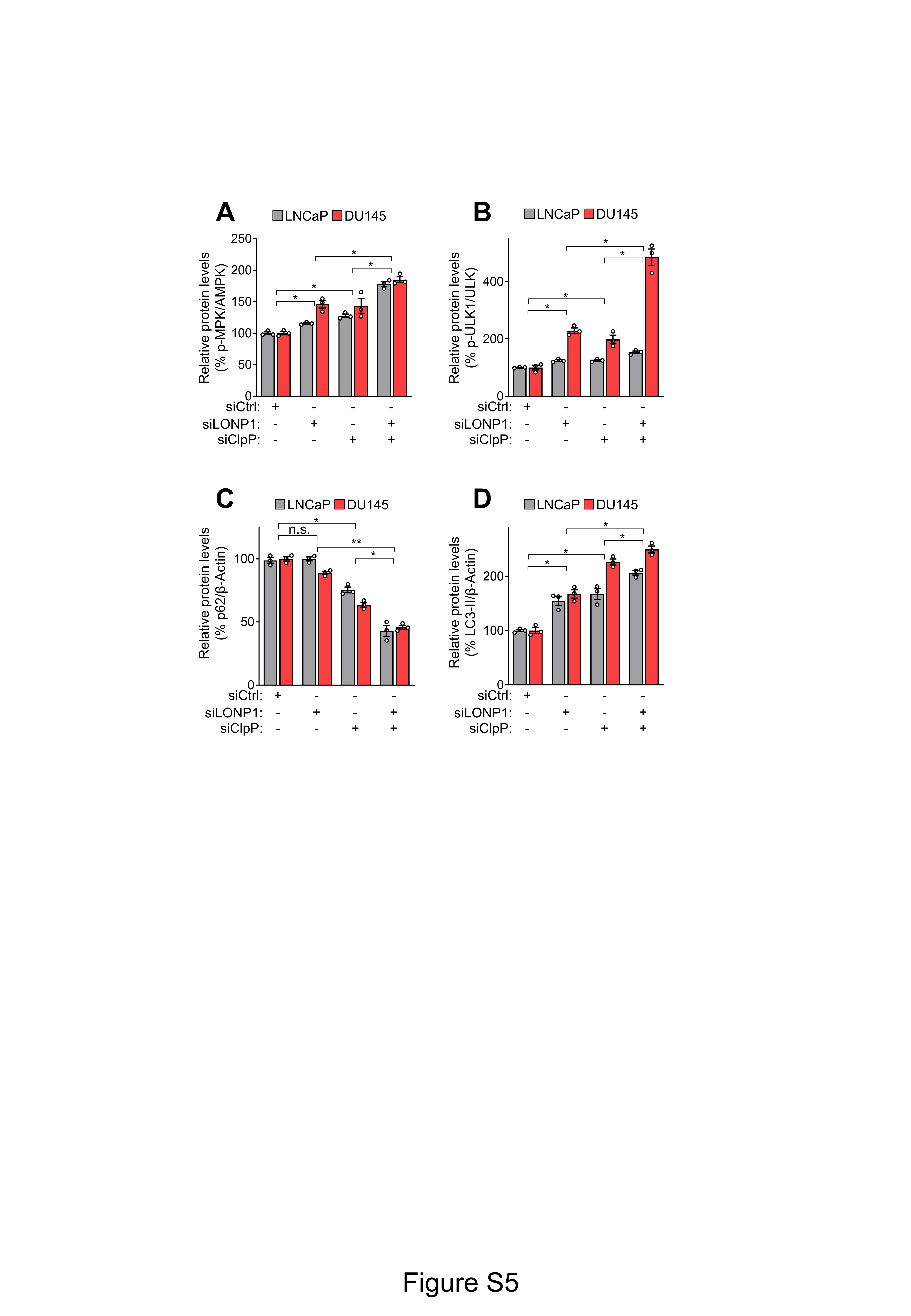

Supplement: Supplementary file 6 — Supplementary Figure 5 [file 41389_2021_306_MOESM6_ESM.tif]

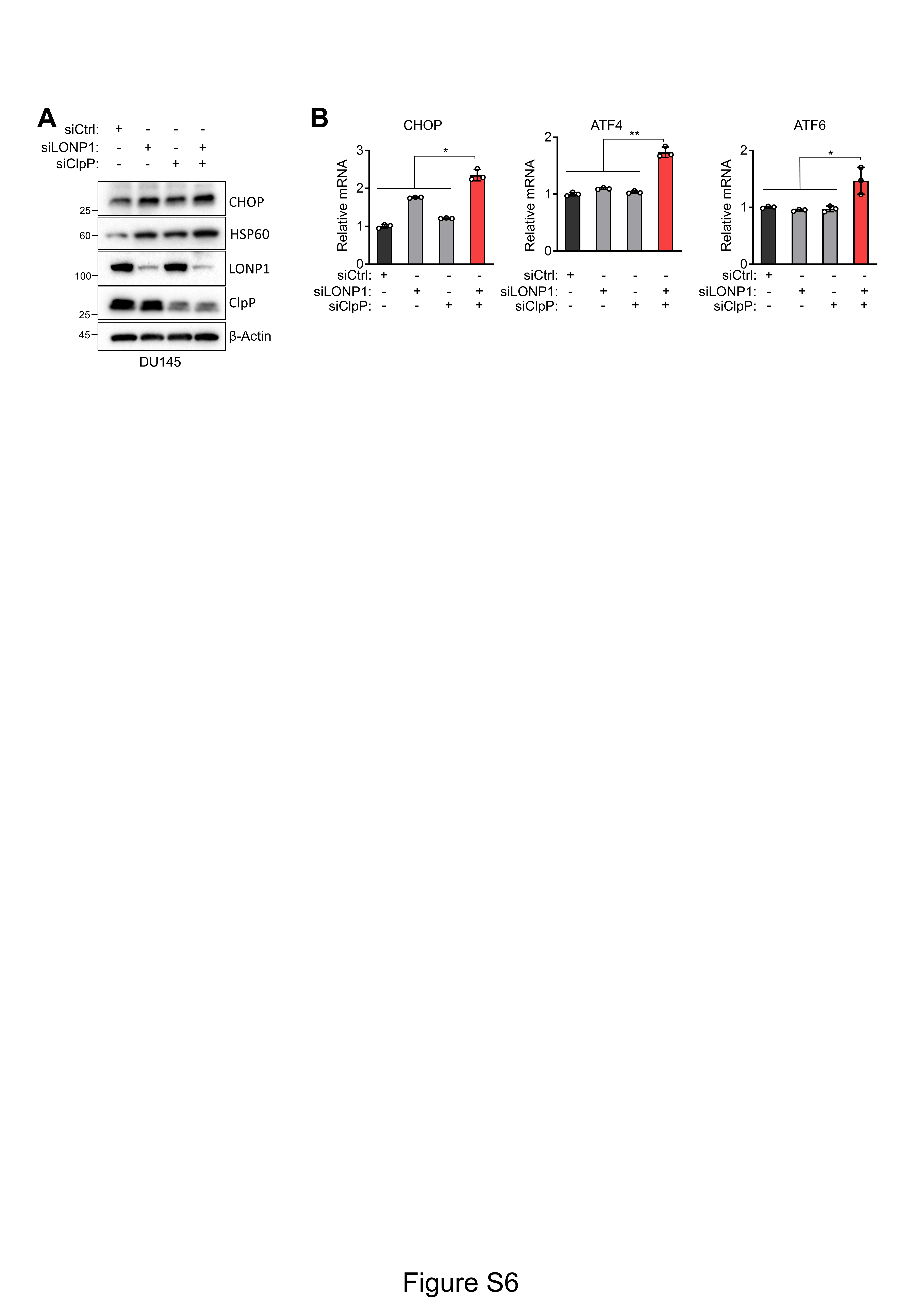

Supplement: Supplementary file 7 — Supplementary Figure 6 [file 41389_2021_306_MOESM7_ESM.tif]

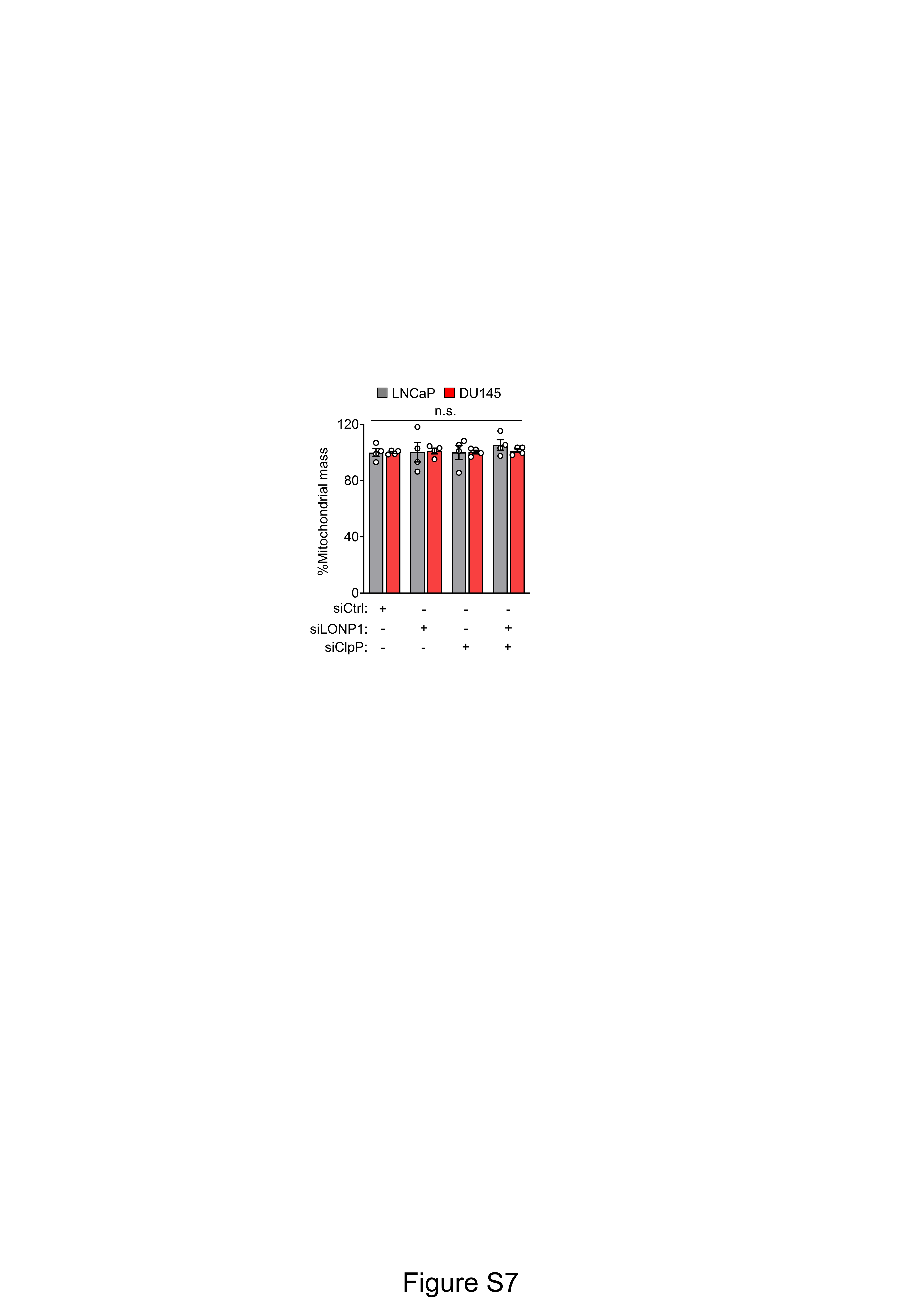

Supplement: Supplementary file 8 — Supplementary Figure 7 [file 41389_2021_306_MOESM8_ESM.tif]

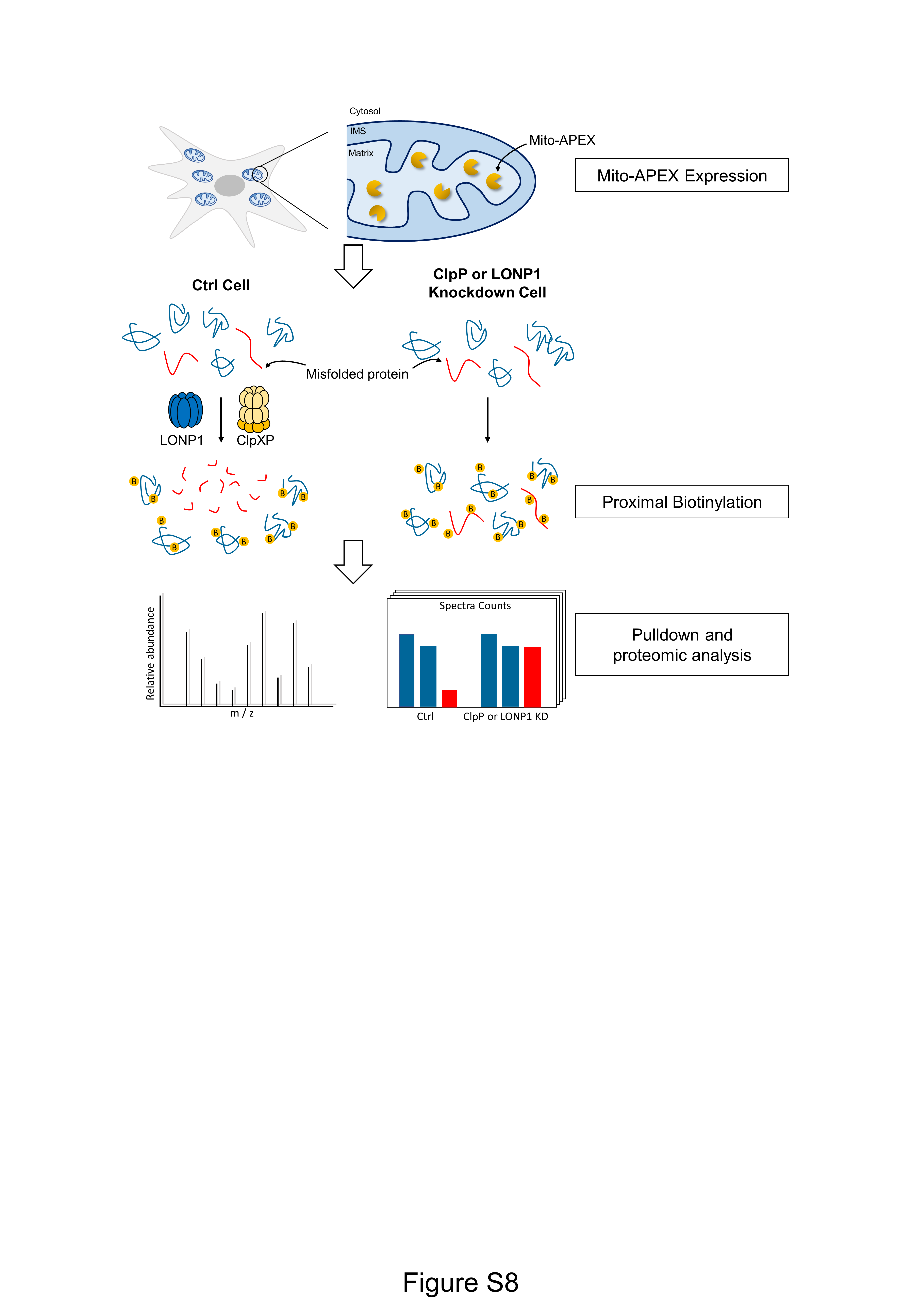

Supplement: Supplementary file 9 — Supplementary Figure 8 [file 41389_2021_306_MOESM9_ESM.tif]

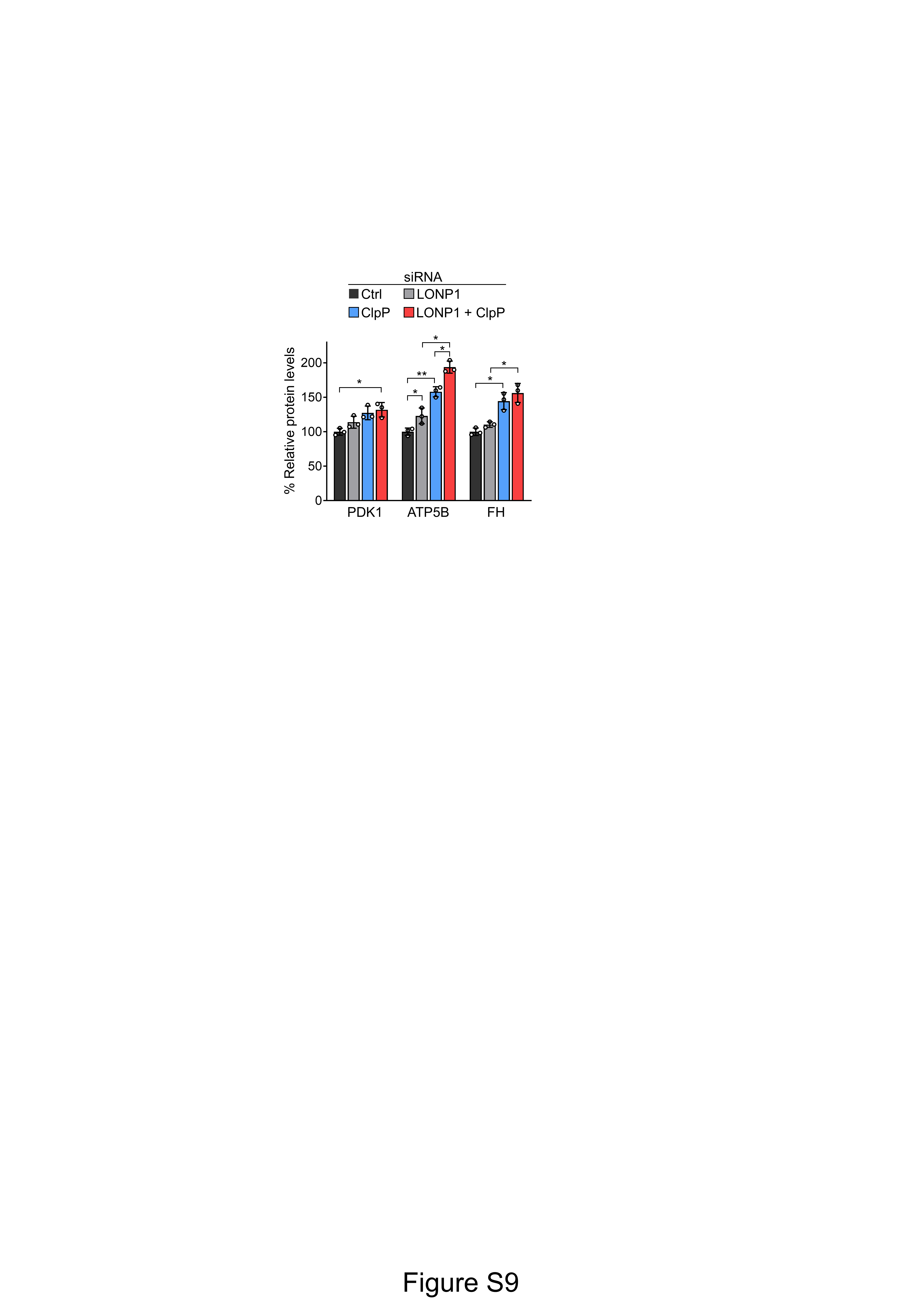

Supplement: Supplementary file 10 — Supplementary Figure 9 [file 41389_2021_306_MOESM10_ESM.tif]

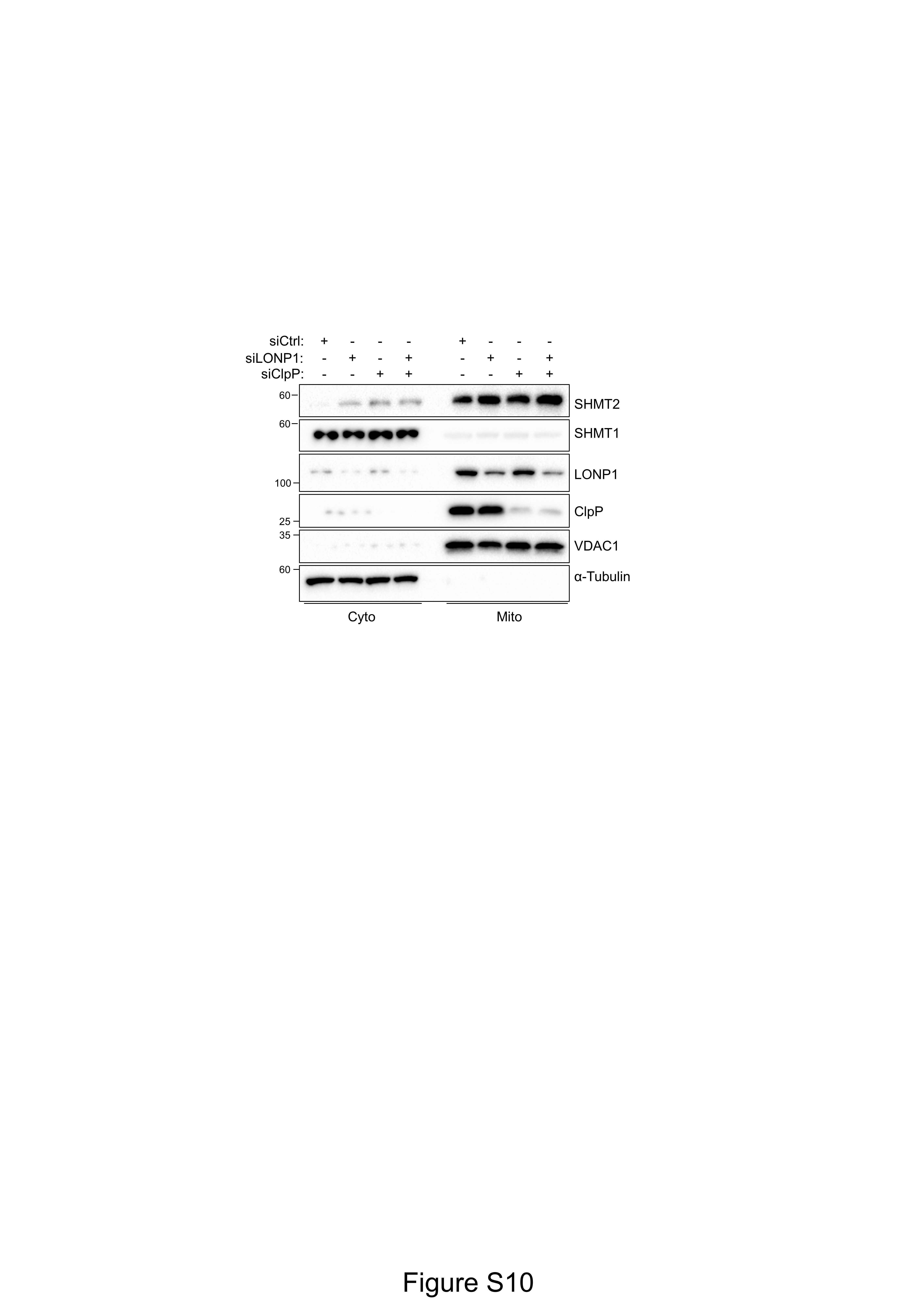

Supplement: Supplementary file 11 — Supplementary Figure 10 [file 41389_2021_306_MOESM11_ESM.tif]

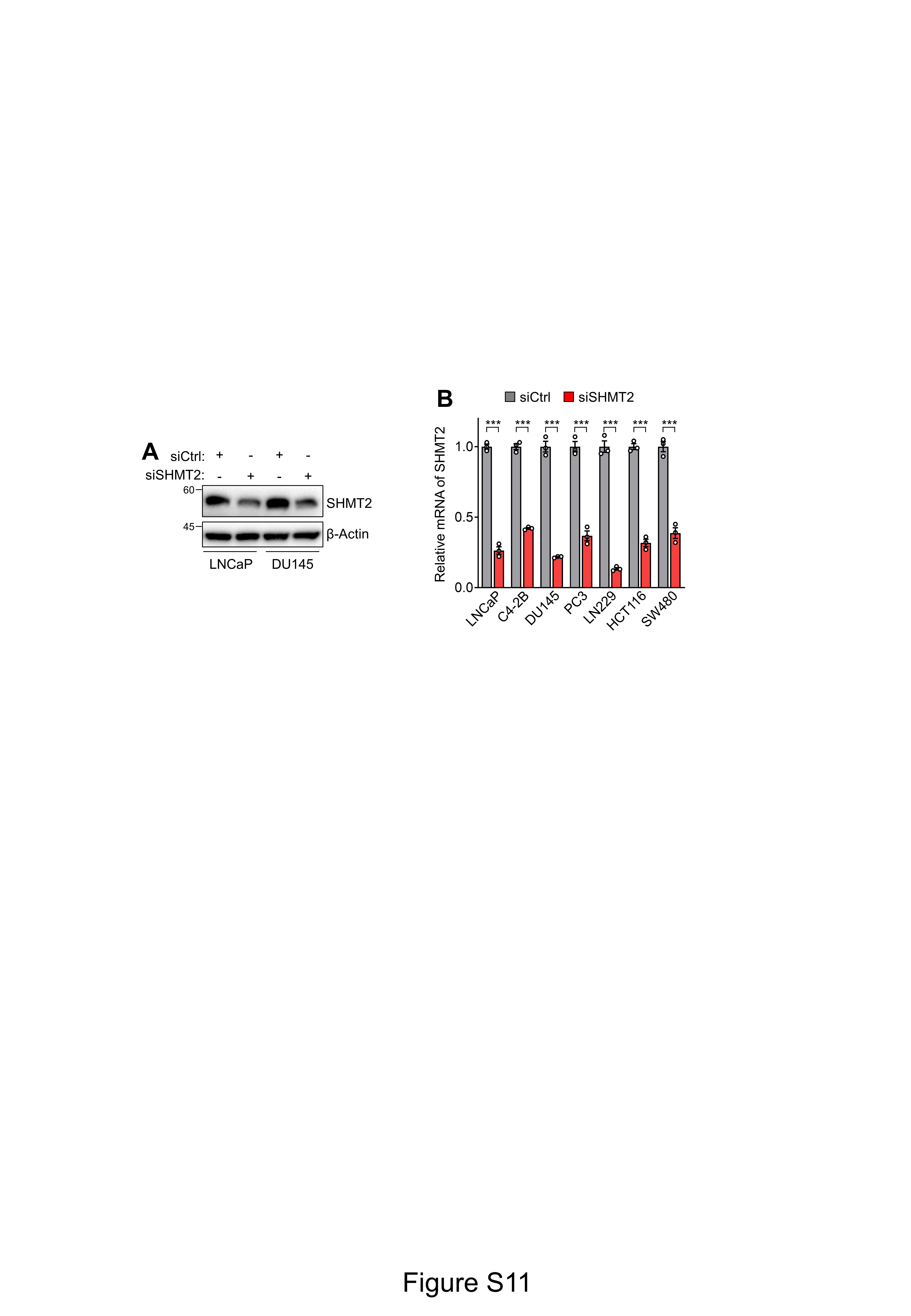

Supplement: Supplementary file 12 — Supplementary Figure 11 [file 41389_2021_306_MOESM12_ESM.tif]
